# Spatiotemporal Variability of Dendroecological Indicators in Pedunculate Oak (*Quercus robur* L.) Tree‐Rings Across Europe in Relation to Species Distribution Models

**DOI:** 10.1111/gcb.70567

**Published:** 2025-10-30

**Authors:** Andrei Popa, Jernej Jevšenak, Marcin Dyderski, Radosław Puchałka, Allan Buras, Ionel Popa, Martin Wilmking, Aleksandra Kalisty, Catalin‐Constantin Roibu, Marcin Jakubowski, Eric Thurm, Martin Šenfeldr, Marko Smiljanić, Ernst van der Maaten, Jan Esper, Edurne Martinez del Castillo, Vaclav Treml, Jan Tumajer, Tzvetan Zlatanov, Roberts Matisons, Gheorghe Florenta, Veronica Florenta, Maksym Netsvetov, Vladislav Grati, Andreas Burger, Karolina Janecka, Saša Kostić, Kamil Pilch, Diāna Jansone, Agnese Liepiņa, Yulia Prokopuk, Oleksandr Sylenko, Mátyás Árvai, Achim Bräuning, Cristina Marques, Martin Häusser, Emil Horváth, Jakub Jeleń, Ryszard Kaczka, Zoltán Kern, Tomáš Kolář, Marcin Koprowski, Sandra Metslaid, András Morgós, Oleksandr Khodosovtsev, Aleksei Potapov, Michal Rybníček, Irena Sochová, Kristina Sohar, Vasyl Budzhak, Ewa Zin, Tassilo Schneider, Wojciech Gil, Marcin Klisz

**Affiliations:** ^1^ National Institute for Research and Development in Forestry “Marin Drăcea” Bucharest Romania; ^2^ Faculty of Silviculture and Forest Engineering Transilvania University of Brasov Brasov Romania; ^3^ Department for Forest and Landscape Planning and Monitoring Slovenian Forestry Institute Ljubljana Slovenia; ^4^ Institute of Dendrology, Polish Academy of Sciences Poznan Poland; ^5^ Department of Ecology and Biogeography, Faculty of Biological and Veterinary Sciences Nicolaus Copernicus University Toruń Poland; ^6^ Centre for Climate Change Research Nicolaus Copernicus University Toruń Poland; ^7^ Land Surface‐Atmosphere Interactions TU Munich Freising Germany; ^8^ Center for Mountain Economy Vatra Dornei Romania; ^9^ Institute for Botany and Landscape Ecology, University of Greifswald Greifswald Germany; ^10^ Forsite Consultants Ltd Prince Albert Canada; ^11^ Forest Biometrics Laboratory, Faculty of Forestry “Stefan Cel Mare” University of Suceava Suceava Romania; ^12^ Faculty of Forestry and Wood Technology Poznań University of Life Sciences Poznań Poland; ^13^ Landesforstanstalt Mecklenburg‐Vorpommern, Department of Forest Planning/Forest Research/Information Systems Research Unit Silviculture and Forest Growth Schwerin Germany; ^14^ Department of Forest Botany, Dendrology and Geobiocoenology, Faculty of Forestry and Wood Technology Mendel University in Brno Brno Czech Republic; ^15^ Chair of Forest Growth and Woody Biomass Production TU Dresden Dresden Germany; ^16^ Department of Geography Johannes Gutenberg University Mainz Germany; ^17^ Global Change Research Institute of the Czech Academy of Sciences (Czech‐ Globe) Brno Czech Republic; ^18^ Department of Physical Geography and Geoecology, Faculty of Science Charles University Prague Czech Republic; ^19^ Institute of Biodiversity and Ecosystem Research Bulgarian Academy of Sciences Sofia Bulgaria; ^20^ Latvian State Forest Research Institute “Silava” Salaspils Latvia; ^21^ Forest Research and Management Institute Chisinau Moldova; ^22^ Moldova State University Chisinau Moldova; ^23^ Institute for Evolutionary Ecology of the National Academy of Sciences of Ukraine Kyiv Ukraine; ^24^ BIOGECO, INRAE, University of Bordeaux Cestas France; ^25^ Institute for Environmental Sciences, University of Geneva Geneva Switzerland; ^26^ Institute of Lowland Forestry and Environment, University of Novi Sad Novi Sad Serbia; ^27^ Dendrolab IBL, Department of Natural Forests Forest Research Institute (IBL) Białowieża Poland; ^28^ Institute for Soil Sciences, HUN‐REN Centre for Agricultural Research Budapest Hungary; ^29^ Institute of Geography, Friedrich‐Alexander‐Universität Erlangen‐Nürnberg Erlangen Germany; ^30^ Chair of Forest and Land Management and Wood Processing Technologies Estonian University of Life Sciences Tartu Estonia; ^31^ Independent Researcher Sárkeresztes Hungary; ^32^ Department of Forest Management, Dendrometry and Economics of Forestry Warsaw University of Life Sciences Warsaw Poland; ^33^ Institute for Geological and Geochemical Research, HUN‐REN Research Centre for Astronomy and Earth Sciences Budapest Hungary; ^34^ CSFK, MTA Centre of Excellence Budapest Hungary; ^35^ Faculty of Forestry and Wood Technology, Mendel University in Brno Brno Czech Republic; ^36^ Global Change Research Institute of the Czech Academy of Sciences Brno Czech Republic; ^37^ Consart Bt. Budapest Hungary; ^38^ Kherson State University Ivano‐Frankivsk Ukraine; ^39^ M.G. Kholodny Institute of Botany Kyiv Ukraine; ^40^ Department of Geography Institute of Ecology and Earth Sciences, University of Tartu Tartu Estonia; ^41^ Falz‐Fein Biosphere Reserve “Askania Nova” Kherson Oblast Ukraine; ^42^ Southern Swedish Forest Research Centre Swedish University of Agricultural Sciences (SLU) Alnarp Sweden; ^43^ Department of Silviculture and Genetics Forest Research Institute (IBL) Sękocin Stary Poland; ^44^ Dendrolab IBL, Department of Silviculture and Genetics Forest Research Institute (IBL) Sękocin Stary Poland

**Keywords:** climate change scenarios, climate–growth relationships, climatic water balance, growth synchrony, range contraction, vapor pressure deficit

## Abstract

Climate is a primary, but non‐stationary, driver of tree growth. Climate change is altering the sensitivity of forest growth to water availability and temperature over time. It is considered that pedunculate oak ( *Quercus robur* L.) will cope with the changing climatic conditions in Europe in the near future. However, while species distribution models project expansion zones, they also identify reductions in occurrence at the dry and warm distribution margins. Whereas species distribution models primarily rely on occurrence data, tree rings—given their long‐term perspective and their use in empirical models—can provide a mechanistic view of forest growth dynamics, including temporally changing climate responses. Increased climate sensitivity and growth synchrony are key dendroecological indicators of tree stress. Here, we used an unprecedented network of 150 *Q. robur* sites (over 3300 trees), covering the full projected range of contracting to persistent areas across Europe, to assess the dendroecological indicators over recent decades in relation to species distribution model predictions. We reveal that oaks in areas projected to experience range contraction exhibited greater sensitivity to current growing season climatic conditions, whereas those in persistence areas responded more strongly to previous season conditions. Growth synchrony among trees was higher in the contraction areas, but showed no significant increasing trend over the last 70 years, as expected from ecotone theory. Temporal shifts in climate sensitivity were stronger for temperature and vapor pressure deficit in the persistence areas, whereas the climatic water balance gained importance in the contraction zones. These findings suggest that *Q. robur* growth is not yet being severely affected by climate change, and that the species is currently coping well with the climate changes, even in regions with projected range contractions, thereby challenging statistically derived scenarios of range shift based on species distribution models.

## Graphical abstract

Our study examined how the pedunculate oak ( *Quercus robur* ) responds to climate change across Europe, utilizing tree rings from over 3300 trees. We found that oaks in drier regions are more affected by current growing‐season conditions, while those in cooler areas respond to the climate conditions of previous growing seasons. Overall, the results suggest that the pedunculate oak is still coping well with climate change, showing greater resilience than predicted by models projecting strong range contractions.

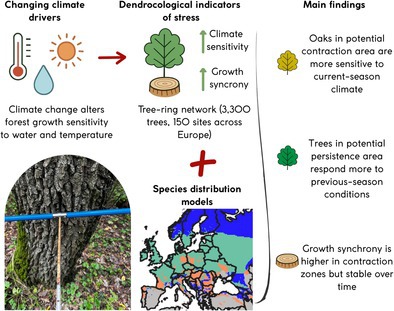

## 1 Introduction

In recent decades, climate change has manifested as a continuous warming trend accompanied by an increased frequency and intensity of extreme weather events (Cook et al. 2020; Spinoni et al. 2018; Zscheischler and Seneviratne 2017). This has severely affected terrestrial ecosystems across the globe (Li et al. 2018). Future climatic scenarios project continuously increasing temperatures (Calvin et al. 2023), making forests vulnerable to future climate extremes (Allen et al. 2015). Climate change may force tree species to adapt to new environmental conditions to varying degrees, which is likely to result in shifts in species distributions (Dyderski et al. 2025; Gougherty et al. 2021).

Tree rings constitute a widely used proxy for secondary tree growth as well as tree resilience to extreme events and drought tolerance (Babst et al. 2017). Climate is one of the most influential limiting factors on tree growth because it directly influences the availability of energy and water resources (Kozlowski and Pallardy 1996). These climate constraints on growth are particularly pronounced in dry (water‐limited) and cold (temperature‐limited) regions, which often correspond to the margins of the species' distribution ranges (Speer 2010). Enhancing the climatic resilience of forest ecosystems and increasing their carbon storage capacity are key priorities of sustainable forestry worldwide (Fetting 2020). Understanding the current and historical responses of trees to climate is essential for predicting how they will respond to environmental changes (Peltier and Ogle 2020) and for forecasting changes in forest compositions due to changing climatic conditions (Buras and Menzel 2019; Chakraborty et al. 2021; Mauri et al. 2022).

Synchronous growth patterns among trees over large areas with comparable environmental settings indicate that macroclimatic variability is a major limiting factor influencing growth (Trouet et al. 2012). Conversely, within‐population synchrony, commonly quantified as a correlation among individual trees in dendroclimatological studies, represents a comprehensive ecological indicator for assessing the degree of environmental stress experienced by forest ecosystems (Tejedor et al. 2020; Wigley et al. 1984). Furthermore, both regional growth synchrony and within‐population synchrony have proven to be effective early warning signals of climate‐induced forest decline (Camarero et al. 2015; Popa, van der Maaten, et al. 2024; Shestakova et al. 2016), although in temperature‐limited environments, ongoing warming trends have been associated with reductions in regional growth synchrony (Shestakova et al. 2019). Consequently, temporal shifts in growth synchrony can be associated with changes in the climate sensitivity of trees (e.g., to temperature and/or water limitations). In forest ecosystems where temperature and moisture limitations interact, trees may alter their sensitivity from one climatic factor to another over time (Árvai et al. 2018; Debel et al. 2021; Leifsson et al. 2023, 2024; Popa, Jevšenak, et al. 2024). Such shifts might represent an adaptation lag in the trees in response to the rapid pace of climate change (Fréjaville et al. 2020). Changes in environmental conditions can trigger a range of tree responses, from persistence through acclimatization to local adaptation (evolution) or, if plasticity is insufficient, to mortality (Bussotti et al. 2015).

Species distribution models (SDMs) are valuable tools for predicting potential changes in species distributions over time, integrating species occurrence data with environmental variables (Elith and Leathwick 2009). Consequently, SDMs are widely employed in ecological studies for diverse purposes, including informing conservation decisions (Guisan et al. 2013), modeling the distribution of invasive species in the context of climate change (Mainali et al. 2015; Puchałka et al. 2023), and economic forest valuation under climate change (Booth 2017; Mellert et al. 2011; Piedallu et al. 2013). The output of SDMs, which identify areas of potential range contraction (current habitats that may become unsuitable), persistence (habitats suitable both now and in the future), or expansion (areas currently unsuitable, but potentially suitable in the future), provides a large‐scale perspective on how specific species' suitable habitats may shift in space under future climatic conditions (Wessely et al. 2024). However, for SDMs, the input data are typically limited to species occurrence combined with current and projected climate data (Dyderski et al. 2025) or, additionally, phenotypic traits (Benito Garzón et al. 2019). The aim of SDMs is to predict fundamental niche maps, usually constrained to climatic niches, which show the range of environmental conditions that a species can potentially inhabit. These maps account for ecological requirements at different stages of ontogeny, indicating areas conducive to survival, development, and reproductive success (Phillips et al. 2006). Recently, SDMs have been widely used to identify alternative species to European trees in response to the risk of decline of some native forest species (Dyderski et al. 2025; Puchałka et al. 2023; Thurm et al. 2018). Rising temperatures may shift tree resource allocation from growth to seed production, even in areas with sufficient water availability (Hacket‐Pain et al. 2025). Therefore, climate change is expected to reduce masting and reproductive success, even in regions where the species' range is projected to expand (Foest et al. 2025). These findings raise concerns about whether the stability of the fundamental niche, as determined by SDMs, aligns with optimal tree growth conditions, suggesting that ecological niche stability does not necessarily reflect favorable conditions for mature trees.

Because climate change has already significantly influenced secondary growth production globally (Babst et al. 2013, 2019), climate–growth relationships provide important information when evaluating tree survival or overall performance. This information can enhance the interpretability of SDM projections. In our study, we introduced a new perspective on the stress‐dominance hypothesis, specifically to test whether SDM projections are already reflected in contemporary growth dynamics, i.e., trees growing in areas predicted to experience a potential range contraction should already exhibit increasing climate sensitivity and growth synchrony. Conversely, in areas where species are projected to persist, trees are expected to exhibit stable patterns in terms of growth rate and synchrony, particularly when trees are well adapted to prevailing conditions. To examine these patterns, we focus on mature, dominant trees because they are the primary elements of forest ecosystems (Ellison et al. 2005) and are determinants of the nutrient cycle, light availability, and regulation of the microclimate of the entire ecosystem (Mueller et al. 2016; Reich et al. 2005; von Arx et al. 2012).

Pedunculate oak ( *Quercus robur* L., hereafter referred to as oak) is an anisohydric species that is considered one of the most promising species for future European forests (Hanewinkel et al. 2013). According to Dyderski et al. (2018, 2025) and Hanewinkel et al. (2013), oak is classed as a ‘winning’ species in Europe, projected to experience a net gain in range as the climate warms. Oaks are generally considered drought‐tolerant species due to their deep root systems, strong hydraulic conductance, and capacity to quickly replace water from their conductive tissues after drought (Martínez‐Vilalta and Poyatos 2023; Martín‐Gómez et al. 2023). Moreover, oaks are ring‐porous species with large earlywood vessels, leading to increased hydraulic conductivity and stomatal conductance, although this feature also makes them vulnerable to cavitation (Peters et al. 2023). Although oak has a core part of its range in the central and eastern parts of Europe, to the best of our knowledge, no pan‐continental study has yet systematically assessed changes in its climate sensitivity using dendroecological indicators. At the European level, especially in the western parts of the continent, the responses of oak to climate (e.g., Bose et al. 2021; Cufar et al. 2014) or extreme climatic events (e.g., Bose et al. 2024; Jiang et al. 2024; Stojanović et al. 2025) have previously been studied on large scales. However, in the eastern regions of its distribution area, most of the studies have been of regional or local extent (e.g., Gričar et al. 2013; Roibu et al. 2020; Sochová et al. 2021, 2024). In this study, we endeavored to test whether SDM projections aligned with the principle of limiting growth factors, particularly given that climate change has already had a significant impact on oak‐dominated ecosystems across Europe (Bose et al. 2021). In particular, we intended to emphasize the observed impacts of climate change on oak productivity in regions projected to become unsuitable for the species' climatic requirements in the near future as an early indication of oak decline.

Accordingly, in this study, we aimed to: (1) identify the key climatic factors influencing oak growth across temperate forest ecosystems in Europe; (2) examine how oak sensitivity to these climatic factors has changed over recent decades; and (3) assess the differences in oak growth synchrony and response to climate in areas under different SDM projections.

Specifically, we hypothesized that oak's responses to climate change would be manifested by:

- *Increasing vulnerability to water availability in contraction areas* (H1): Oaks in contraction areas of their range are mainly water‐limited, and as droughts become more frequent, we can expect their vulnerability to increase, reinforcing projections of future range contraction.
- *A decrease in its climate sensitivity in persistence areas* (H2): Sites projected to persist under future climate experience less pronounced climate limitations, reflected in weaker climatic constraints and lower growth synchrony compared to sites located in areas projected to undergo range contraction.
- *Varying temporal shifts in climate sensitivity in its contraction and persistence areas* (H3): Due to recent climate change, particularly global warming, we anticipate more pronounced changes in oak climate sensitivity in its contraction areas, whereas shifts in persistence areas are expected to occur at lower rates.

## 2 Material and Methods

### 2.1 Study Area and Tree‐Ring Data

**FIGURE 1:**
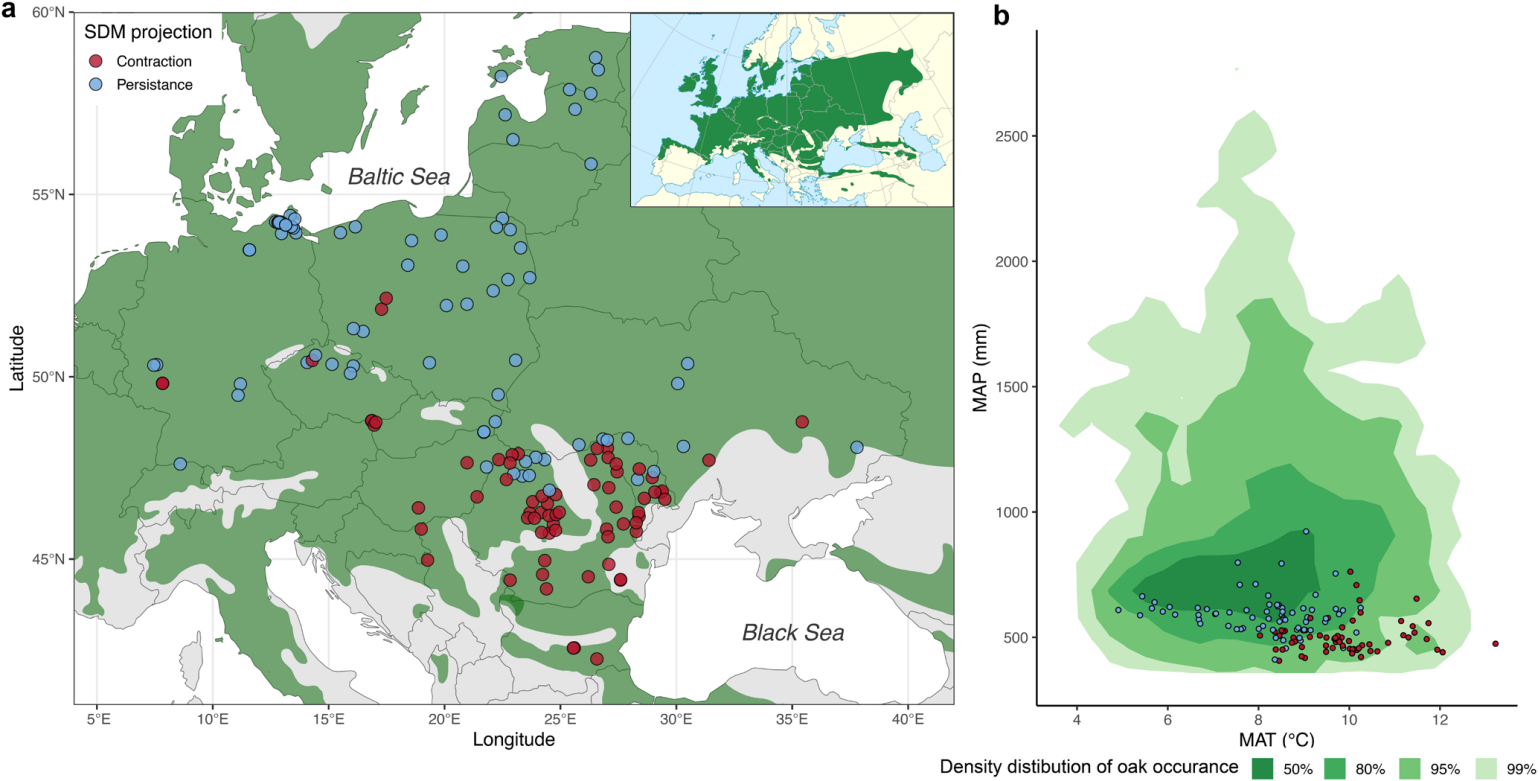
(a) Map of the study area showing site locations (points), with colors indicating the species distribution model (SDM) projection under climate scenario SSP370 (Dyderski et al. 2025). The shaded green area depicts the distribution of *Q. robur* in the study area, with the inset showing the occurrence at the European level (Caudullo et al. 2017). (b) Mean annual temperature (MAT) and mean annual precipitation (MAP) for the study sites, the colored areas representing the kernel density levels at 50%, 80%, 95%, and 99% for *Q. robur* occurrence in Europe, and the points representing the study plots colored accordingly to the SDM projection (see panel a). Map lines delineate study areas and do not necessarily depict accepted national boundaries.

The study area spanned Central and Eastern Europe, encompassing the core region and southernmost limits of the *Q. robur* distribution in Europe (Figure 1a). The network used for our study was distributed along a latitudinal gradient of 16°30′, ranging from 42°15′ N in central Bulgaria to 58°45′ N in northern Estonia. It also spanned a longitudinal gradient of 30°20′, extending from 7°27′ E in western Germany to 37°47′ E in eastern Ukraine. Across the network, the mean annual temperature ranged from 4.9°C in Estonia to 13.2°C in Bulgaria, while the annual precipitation ranged from 410 mm in Romania to 920 mm in Germany.

The compiled network included tree‐ring width (TRW) data from 3304 oak trees across 150 sites. Most of the TRW data was collected recently, with more than 90% of the chronologies sampled in 2018 or later (Figure S2c). Each study plot represented an oak‐dominated mature tree stand. In each plot, 10 to 75 trees were sampled, with more than 76% of the plots comprising 20 or more trees, with an average of 22 trees per plot. From each tree, one or two increment cores were collected, processed, and measured following standard dendrochronological procedures, which included drying, surface preparation, and TRW measurements using either high‐resolution images or specialized measuring systems, such as Lintab (Speer 2010). At the plot level, before inclusion in the final database, individual TRW series were statistically cross‐dated using COFECHA software (Holmes 1983) and validated by standard statistical parameters used in dendrochronological studies (Speer 2010). To emphasize high‐frequency climate signals, size‐ and age‐related trends were removed by detrending individual tree‐ring series using a 30‐year smoothing spline (Cook and Kairiukstis 1990). A TRW index (RWI) series was calculated as the ratio between the measured series and the fitted spline function. Plot‐level chronologies were computed by averaging the individual series using a biweight robust mean that minimized outlier impacts (Cook and Kairiukstis 1990). Finally, an autoregressive model was applied to eliminate the influence of autocorrelation, also known as pre‐whitening (Cook 1985). These pre‐whitened site chronologies were used for the subsequent analyses. The detrending process and chronology‐building were performed using the R package ‘dplR’ (Bunn 2008).

### 2.2 Climate Data

For each plot, we retrieved gridded daily climate data, including the mean, minimum, and maximum temperature, precipitation, and relative humidity. We used the E‐OBS v29.e database, with a resolution of 0.1 × 0.1°, covering the period 1950–2023 (Cornes et al. 2018). We also obtained the bioclimatic variables for 1981–2010 from the CHELSA database at a resolution of 1 × 1 km (Brun et al. 2022), which were used in the modeling part of our study (see Section 2.6). Climatic data for the climate space diagram (Figure 1b) were obtained from the gridded database CRUTS4.08 (Harris et al. 2020).

Using the daily mean temperature (*T*_mean_) and relative humidity (Rh), we calculated the vapor pressure deficit (VPD) at the daily level in kilopascals Equation (1).

(1) $$\mathrm{VPD}=0.6108*\exp^{17.269*\frac{T_{\text{mean}}}{T_{\text{mean}}+237.3}}*\left(1-\mathrm{Rh}\right)$$

The climatic variables were aggregated into monthly values by calculating the means of the temperature and VPD and the sum of the precipitation. Then we calculated the monthly potential evapotranspiration (PET) using the Hargreaves–Samani method (Hargreaves and Samani 1985), as described in Equation (2), where *T*_mean_, *T*_min_, and *T*_max_ depict the mean, minimum, and maximum temperatures, and RA is the extraterrestrial radiation (in megajoules per square meter per day), calculated from the latitude, solar declination, and day of the year (Allen et al. 1998).

(2) $$\mathrm{PET}=0.0023\cdot \mathrm{RA}\cdot \left(T_{\text{mean}}\right)\cdot \sqrt{T_{\max}-T_{\min}}$$

The climatic water balance (CWB) was then calculated as the difference between the precipitation and PET. The long‐term variability of the mean annual temperature and annual precipitation at the plot level and for the entire network is shown in Figure S2. To calculate the PET, we used the ‘SPEI’ R package (Beguería et al. 2017).

### 2.3 Climate–Growth Relationships and Their Variation Across Geographic and Climatic Space

To assess oak's climate sensitivity, we first divided the previous and current years of ring formation into three seasons: (1) the previous growing season from June to October in the previous year; (2) the dormancy season from the previous November to the current March; and (3) the current growing season from April to September (Chen et al. 2022). We selected the period from June to October for the previous growing season to capture potential legacy effects on the following year's growth, as early‐season conditions primarily influence current‐year wood formation and post‐June/July conditions potentially exert stronger impacts on growth in the subsequent year (Gao et al. 2018; Huang et al. 2018). For each of these seasons, we calculated the Pearson's correlation coefficients between the RWI and the monthly or periodical (an aggregation of 2–5 months) climate factors (i.e., mean temperature, CWB, and VPD). The significance of these correlations was assessed using a bootstrap approach with 1000 replications (Efron and Tibshirani 1986). The climate–growth relationships were assessed using the function *monthly_response* from the ‘dendroTools’ R package (Jevšenak 2020; Jevšenak and Levanič 2018). To account for the temporal shifts in climate sensitivity (TSCS), as defined in Popa, Jevšenak, et al. (2024), these correlations were calculated for the entire study period and the two subperiods of 1950–1985 (defined as the early period) and 1986–2023 (defined as the late period). Differences among the distribution of correlations were tested using the Kolmogorov–Smirnov test (Massey Jr. 1951).

**FIGURE 2:**
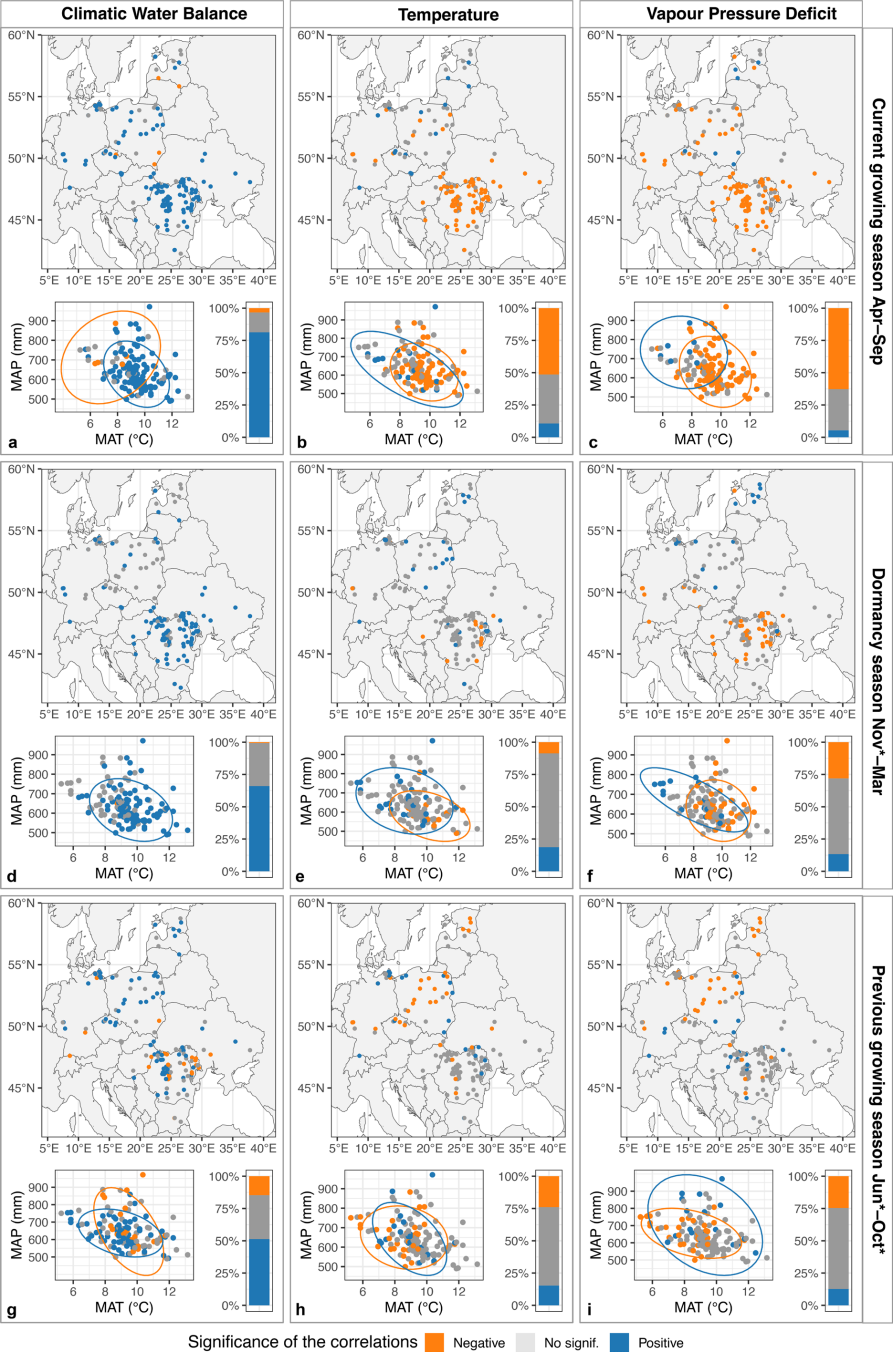
The spatial variability of oak sensitivity to climate across different seasons (rows) and climatic parameters (columns) (a–i). The variability of the correlation coefficients is shown in relation to mean annual temperature (MAT) and mean annual precipitation (MAP), and the percentage of significant correlations. The colors indicate the significance of the correlations, with circles highlighting the clusters of plots facing the same climate sensitivity in terms of positive, negative, or no significance. Asterisk (*) represents the month in the year preceding ring formation (no asterisk = current year of ring formation). Map lines delineate study areas and do not necessarily depict accepted national boundaries.

To test the first hypothesis, we extracted the highest correlation coefficient for each site across the three predefined seasons, considering the entire study period. To explain the variability of the influence of key climatic parameters (mean temperature, CWB, and VPD) on oak growth, we fitted linear models between the correlation coefficients and longitude and latitude (Figures S3 and S4). We then categorized these correlations into negative, positive, and insignificant, and grouped them into clusters based on their significance (Figure 2). Finally, we calculated the percentage of sites with significant correlations (*p* < 0.05) (Figure 2), as well as the median across all correlations (separated for positive and negative), regardless of significance, independently for each season (Figure S5).

### 2.4 Species Distribution Model for Oak

The SDM suggested that oak will experience divergent trends in its potential range in Central and Eastern Europe (Dyderski et al. 2025). To address the second hypothesis, we focused on the SDM projections under the Shared Socioeconomic Pathways (SSP) 370 climate scenarios for 2061–2080, provided by Dyderski et al. (2025). To test the robustness of our conclusions and the effects of stronger and weaker climate changes, we also assessed the SSP245 and SSP585 scenarios. We focused on the SSP370 scenario because it represents a medium to high level of change, bridging the intermediate (SSP245) and pessimistic (SSP585) scenarios (Figure S1). Based on the SDM projections, oak in the southern part of its distribution in Europe is expected to experience a range contraction in the future, affecting 71 of the sites in our network (Figure 1). Conversely, in large parts of Central Europe, oak is projected to persist within its suitable range in our network. To test the second and third hypotheses, we first compared climate sensitivity and growth synchrony between both groups (H2) and then assessed the temporal trends of these two dendroecological indicators (H3).

### 2.5 Modeling Changes in Growth Synchrony

To address the second hypothesis (H2), we assessed the growth synchrony using two methods. Firstly, we focused on the within‐population synchrony—that is, the coherency in individual tree growth patterns at the plot level (Tejedor et al. 2020). To do this, we used the average correlation of all pairwise‐RWI series within a specific plot (i.e., the *rbar*) (Wigley et al. 1984). We calculated the running *rbar* values using a 20‐year moving window and aggregated individual plot‐level means based on the SDM projections, i.e., for potential persistence and contraction areas. Secondly, in order to assess the synchrony patterns (â_C_) across spatially larger areas, we employed a variance–covariance model using the homoscedastic variant of the full model defined by Shestakova et al. (2014), with the SDM projection as a grouping variable. The model was applied for the previously defined early and late periods (see Section 2.3). In the case of within‐population synchrony, the *rbar* values ranged from 0 = no synchrony between individual trees to 1 = fully synchronized growth dynamics (Wigley et al. 1984). For the synchrony patterns (â_C_), the values ranged from 0 = no regional synchronization between study plot chronologies to 1 = fully regional synchronization (Shestakova et al. 2014).

### 2.6 Modeling Temporal Shifts in Climate Sensitivity

To address the third hypothesis, we first identified all significant correlations (*p* < 0.05) across different time intervals (months or cumulative months) for the entire study period. Then, focusing only on the selected intervals, we compared the distributions of the correlations between the early (1950–1985) and late (1986–up to 2023) periods. To quantify the temporal shifts in climate sensitivity, we calculated the differences between the absolute correlation values between the late and early periods, respectively (hereafter referred to as delta, *Δr*) (Jevšenak et al. 2024). Finally, we applied the pairwise Wilcoxon test to assess the differences in the delta coefficients (indicators of TSCS) between the regions of range contraction and persistence.

To investigate how oak sensitivity to climate varies across time and space, and to identify those climatic parameters with the strongest influence on radial growth, we fitted linear mixed‐effects models (Zuur 2009). The dependent variable was climate sensitivity, expressed as a correlation coefficient, with the explanatory variables being: (1) SDM projection (categorical variable—range contraction or range persistence) as a spatial variable; (2) the climatic space of the network—bioindicators (average values for the entire study period) with the highest relative importance in the SDM (Dyderski et al. 2025), such as BIO18—mean monthly precipitation in the warmest quarter, BIO9—mean daily air temperatures in the driest quarter, BIO10—mean daily air temperatures in the warmest quarter, and BIO11—mean daily air temperatures in the coldest quarter; (3) variability in time (categorical variable—early and late periods); and (4) stand mean age (based on the mean age of the analyzed trees approximated from the number of tree‐rings measured in individual cores) to account for the potential variability caused by the different ages of the analyzed stands. Additionally, we considered the interaction between the SDM projection and time periods (early and late). As random effects, we used the season and the site code. Linear mixed‐effects models were fitted using the ‘lme4’ R package (Bates et al. 2009).

We initially fitted a model for climate sensitivity (correlation coefficients) to each of the three climatic variables (CWB, mean temperature, and VPD) independently, using all correlations in early and late periods. To assess the robustness of our findings, we tested models for climate sensitivity fitted to each of the climate parameters, focusing on correlations in early/late periods from intervals (monthly or periodic assessment of climate sensitivity, see Section 2.3 for more details) where significant correlations were observed throughout the entire study period (Table S2). To examine the general variability in the climate sensitivity of oak, we fitted a linear mixed‐effects model similar to those described above, but introducing climate parameters as the fixed effects. Model performance was assessed using several diagnostic plots: the residuals‐versus‐fitted plot to evaluate the linearity assumption, the normal Q‐Q (quantile‐quantile) plot to assess whether residuals follow a normal distribution, the scale‐location plot to assess the homoscedasticity, and the variance inflation factor to detect potential collinearity issues (O'brien 2007; Schützenmeister et al. 2012). These diagnostic plots were generated using the function *check_model*() from the ‘performance’ R package (Lüdecke et al. 2021).

To illustrate the effects of each climatic parameter, the influence of the early and late periods, and their interactions with the SDM projection on oak climate sensitivity, we calculated the marginal effect at the mean (MEM) using the ‘emmeans’ R package (Lenth 2025), and assessed the difference between periods using Tukey's posteriori test (Abdi and Williams 2010).

## 3 Results

### 3.1 Geographical Variability of Oak Sensitivity to Climate

CWB showed a consistently positive relationship with RWI across all three seasons (i.e., current and previous growing seasons and dormant period). The highest correlations between the CWB of the current growing season and the RWI were positive and significant in over 75% of the sites, while during the dormancy period, CWB positively correlated with oak growth in more than 65% of the plots (Figure 2d). The influence of the CWB from the current and dormancy seasons on the oak RWI exhibited a pronounced latitudinal gradient, decreasing from the southern to northeastern sites (Figure 2a,d, and Figure S3). The CWB from the previous growing season induced a divergent pattern to oak radial growth, with a clear overlap occurring between the two clusters of plots with positive versus negative relationships (Figure 2g, bottom).

The temperature and VPD during the current growing season were more influential climate drivers of oak growth (more significant correlations) than the conditions from the previous growing season (Figure 2). Moreover, a strong latitudinal gradient was observed in temperature correlations from the current growing season, with the southern (warmer) sites exhibiting a negative relationship, whereas the northern (colder) sites showed a positive temperature effect (Figure S3). Regarding the influence of VPD during the current growing season on oak growth, we observed a less pronounced latitudinal gradient than for temperature, with the southern sites (corresponding to the contraction area) being more negatively affected by high temperatures than the northern sites (corresponding to the persistence area). However, a larger proportion of sites (66%) demonstrated negative and significant correlations between VPD and RWI (Figure 2c). For the VPD from the previous growing season, significant negative correlations were observed at approximately 25% of the sites, suggesting a weaker impact of high VPD from the previous growing season on growth in the following year. In terms of the longitudinal gradient, no significant trend was detected from west to east for CWB or VPD, although a west–east gradient was observed for temperature correlations during the current growing season (Figure S4). When considering all correlations, regardless of their significance, the median values followed a similar pattern to the one observed when focusing exclusively on the highest seasonal correlations (Figure S5).

### 3.2 Response of Oak to Climate in Regions Under Range Contraction and Range Persistence

**FIGURE 3:**
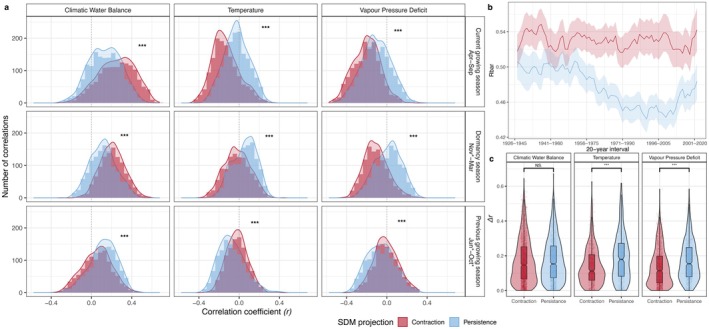
(a) Distributions of the correlation coefficients in areas of contraction and persistence across different seasons for the entire study period. (b) Mean growth synchrony (*rbar*) in the SDM projections. Shaded areas represent the standard error; the *x*‐axis presents the interval for which the *rbar* was calculated, indicated by the end of the reference window. (c) Temporal shift in climate sensitivity, where *Δr* depicts the absolute difference in the correlations between the late and early periods, respectively. From the Wilcoxon test, *** represents *p* < 0.001. In panel (a), the asterisk (*) represents the month in the year preceding ring formation (no asterisk = current year of ring formation).

The distribution of all correlations, regardless of their significance, revealed notable differences between sites projected to experience a range contraction and those in areas of potential range persistence (Figure 3a). The Kolmogorov–Smirnov test revealed significant differences in the distributions of correlation coefficients between sites located in potential contraction versus persistence areas (all *p* < 0.01). During the current growing season, contraction‐area sites showed stronger negative sensitivity to temperature and VPD, and stronger positive sensitivity to CWB, relative to persistence‐area sites. In the dormancy period, contraction‐area sites also exhibited stronger growth limitations linked to CWB. By contrast, temperature and VPD effects during dormancy followed the opposite pattern: persistence‐area sites displayed more positive correlations between RWI and these variables. However, during the previous growing season, the oaks in persistence areas were more climate‐sensitive, with stronger negative correlations between growth and both temperature and VPD, and stronger positive correlations with CWB, suggesting a stronger carry‐over effect from the previous growing season for oaks in the areas of range persistence.

The within‐population synchrony (*rbar*) showed no obvious temporal trend in the contraction area (Figure 3b). In the persistence area, however, the *rbar* values were generally lower and also showed a progressive decrease over time by the 1980s. However, in the last three decades, we identified a gradual increase in *rbar* values in the persistence area. The regional growth synchrony between sites in both periods (early and late) was notably higher (â_C_ > 0.29) in areas projected to experience a potential range contraction than in sites in areas of projected range persistence (â_C_ < 0.14) (Figure S9). Moreover, the regional growth synchrony between the early and late periods slightly increased.

Under the SSP585 scenario, we found similar patterns to the ones presented for SSP370 (Figure S7). However, the results obtained for the intermediate scenario (SSP245) suggest different responses because the number of sites in the persistence and contraction areas was unbalanced (Figure S6).

### 3.3 Temporal Shifts in Oak Climate Sensitivity in Areas of Range Contraction and Range Persistence

In areas experiencing a potential range contraction, there were stronger correlations between the current‐season CWB and RWI in the late period than in the early period (Figure S10). For the other seasons and sites from the persistence area, no notable differences were observed between the early and late periods, except the dormancy period, when CWB showed stronger correlations with oak growth in the late period. Overall, no significant differences were found in temporal shifts of the sensitivity of oak to CWB (∆r) when comparing the two SDM areas (Figure 3c).

For the sites in areas projected to experience range persistence, we observed an increased correlation between the RWI and temperature during the dormancy period (Figure S10). Similarly, temporal changes in the previous‐season temperature effects on the RWI were more frequent in oaks from the persistence area, but in the direction of weaker high‐temperature constraints on growth patterns. By contrast, oaks in the contraction areas exhibited smaller changes in temperature sensitivity over time compared to those in the persistence areas (Figure 3c). In the case of VPD, oak from the potential contraction area faced a decrease in the sensitivity for the VPD condition during the current growing season. However, the temporal shifts in VPD sensitivity (∆r) were stronger in the persistence area (Figure 3c).

**FIGURE 4:**
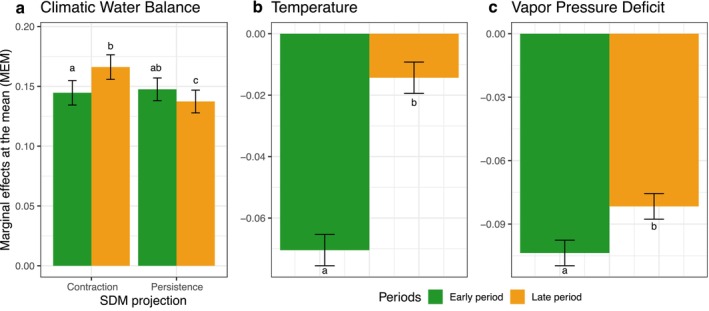
Marginal effects at the mean of the study periods (early and late) on oak sensitivity to (a) climatic water balance, (b) mean temperature, and (c) vapor pressure deficit in relation to the SDM projections (potential range contraction or persistence). Note that the models for (b) temperature and (c) vapor pressure deficit sensitivity did not include the SDM projections as a significant fixed effect (see Table S1). Error bars show ± one standard deviation. Letters indicate the significant differences between the categorical predictors based on Tukey's posteriori test.

Based on the mixed‐effects model, oak sensitivity to the CWB was mostly affected by the period (i.e., early or late), climate space of the network (i.e., BIO18—mean monthly precipitation amount of the warmest quarter, BIO9—mean daily mean air temperatures of the driest quarter, BIO10—mean daily mean air temperatures of the warmest quarter), and the interactions between SDM projection and period (Table S1). The marginal effects of the mean revealed a statistically significant difference during the late period (Figure 4), indicating that oak sensitivity to the CWB has changed over time.

For temperature, the model identified fewer bioclimatic indicators (site climatology) as key drivers of oak sensitivity (only BIO10—the mean air temperatures of the warmest quarter) (Table S1). SDM projection emerged as a significant factor for oak sensitivity to temperatures (*p* = 0.012) for the model incorporating only significant correlations in the early and late periods. Moreover, the explanatory power of the fixed effects was relatively higher (Table S2). We observed a significant difference in the marginal effect of the time period on oak sensitivity to temperature (Figure 4), indicating a change over time in oak sensitivity to temperature, regardless of the SDM projection. The model for VPD sensitivity identified only the mean daily air temperature of the coldest quarter (BIO11) and the period as significant drivers of oaks' sensitivity to this climatic factor. The marginal effect for VPD differed significantly between the periods (Figure 4). All fitted models met the validation assumptions (Figure S8).

The model incorporating the climatic parameters as fixed factors demonstrated a higher explanatory power based on the fixed effects (*R* ^2^_m_ > 0.28), outperforming those models fitted for individual climatic parameters (Table S3). The marginal effects of the mean for the climatic parameters from each period in relation to the SDM projection exhibited similar patterns to those observed for the individual models (Figure S12).

## 4 Discussion

Our study revealed that oak growth is primarily constrained by water availability (the CWB in particular) and by energy dynamics, including the inhibitory effects of elevated temperatures and increased VPD. These limitations were most pronounced in the central part and at the southern edges of the oak's distributional range in Europe. While climate change has well‐documented effects on forest ecosystems (Allen et al. 2015) and has driven shifts in the climatic drivers of tree growth globally (Babst et al. 2019), our findings reveal a more nuanced and regionally variable pattern. Specifically, we observed higher growth synchrony at the tree level in the study plots from the expected contraction zone, and lower, progressively decreasing synchrony in the persistence zone after the 1970s, followed by a slight increase in the last few decades, potentially indicating emerging changes in tree growth patterns. Moreover, the fluctuations in oak climate sensitivity exhibited significant spatial variability across the study region, underscoring the interplay of environmental and climatic factors in driving the growth dynamics.

### 4.1 Oaks Show Stronger Drought Limitation in the Potential Range Contraction Areas

Our results show that the CWB from the current and dormancy seasons was one of the strongest climatic factors driving oak growth in Central and Eastern Europe, generally with a stronger effect in warmer and drier sites (i.e., mostly contraction sites; Figure 2 and Figure S3). Similar findings of strong relationships between oak radial growth and precipitation or CWB during winter and current spring–summer have been reported from Western Europe across large areas, albeit at a lower intensity than in *Quercus petraea* (Matt.) Liebl. (Bose et al. 2021). Furthermore, multiple studies have indicated a positive relationship between oak growth and summer precipitation (e.g., Friedrichs et al. 2008; Nechita et al. 2019; Netsvetov et al. 2017; Sochová et al. 2024). In particular, Basu et al. (2024) and Roibu et al. (2020) indicated that monthly precipitation from March to June correlated with latewood width. In Central and Eastern Europe, mainly at latitudes below 50°, we observed strong correlations (up to *r* = 0.6) between oak radial growth and CWB during the dormancy season. Our results correspond with the findings reported by Bose et al. (2021) for northern France and Germany.

Snow quantity plays a significant role in restoring soil water storage in spring, and any changes in this hydroclimatic variable can lead to a significant impact on terrestrial ecosystems, including forests (Barnett et al. 2005). Allen et al. (2019) reported that oak primarily relies on winter precipitation during the first part of the growing season rather than, as might be expected, spring precipitation. Consequently, as previously demonstrated, the secondary oak growth, particularly earlywood width or earlywood anatomical parameters, positively correlates with the amount of precipitation received in the previous autumn and winter (Gonzalez and Eckstein 2003; Matisons and Brūmelis 2012; Scharnweber et al. 2020). In our study, we focused only on total TRW, and so we likely captured the combined climate signal of both earlywood and latewood. However, because the latewood dimensions contribute the most to the annual TRW of ring‐porous species (Sass‐Klaassen et al. 2011), our findings agree with those of other studies that showed the climate sensitivity of oak latewood (Kern et al. 2013; Nechita et al. 2019; Puchałka et al. 2016).

In warmer and drier regions, we observed a strong negative relationship between current‐year temperature and oak growth. Although a cluster with negative and positive temperature effects overlapped to a great extent (Figure 2b), we observed a pronounced latitudinal gradient, with negative correlations in the southern parts of the distribution (Figure S3). Similar temperature effects have previously been reported for oak stands in Eastern Europe (Cufar et al. 2014; Nechita et al. 2017; Netsvetov et al. 2021). However, Bose et al. (2021) found no negative effects of summer temperature, with the exception of one site in northern Germany. A likely explanation for these differences is the effect of continentality influencing sites in Eastern Europe. Temperatures from previous growing seasons or dormancy periods had little influence on the oaks' radial growth. The negative effects of winter temperatures could be attributed to lower temperatures causing deeper frost levels, which can delay thawing processes in spring and, consequently, the onset of cambium activity (Hu et al. 2010). Notably, only 25% of the plots exhibiting negative temperature effects were located at higher latitudes.

Previous research has shown that VPD, an indicator of atmospheric dryness, can be a strong driver of tree growth, including in oaks (Tumajer et al. 2022). Nevertheless, VPD is still less commonly incorporated into assessments of tree climate sensitivity based on radial growth patterns, compared to other climate parameters, such as temperature, precipitation, CWB, or soil moisture availability indices (but see, e.g., Babst et al. 2019; Marchand et al. 2020; Trotsiuk et al. 2021; Voelker et al. 2014). In the context of climate change and the increasing frequency and intensity of heatwaves, it is essential to understand and quantify the impacts of increased VPD on tree growth (Sanginés De Cárcer et al. 2018). Our results show that, in more than 65% of the plots, oak growth was significantly negatively correlated with VPD in the current growing season, in 10% more cases than with temperature, especially in the northern part of the study area. Thus, VPD should be more frequently considered as an influencing factor on tree growth because stem water balance, which is related to radial growth, is often more strongly related to transpiration than to the limitation of water uptake by the root system (Tumajer et al. 2022; Zweifel et al. 2005, 2016). Notably, we observed a clear separation of plots where VPD negatively affected radial growth (i.e., drier and warmer sites) from those where this relationship was weak or absent (moist sites). Our results also indicate a positive correlation between the VPD during dormancy periods and the RWI, an aspect that was unexpected because oaks do not have any leaves during dormancy. A possible explanation may emerge from the fact that temperature and VPD are correlated, so in this case, this may be a temperature effect.

The positive relationship between oak growth and CWB aligns with the negative correlations between RWI and temperature and VPD. An increased atmospheric water demand (i.e., higher VPD values) leads to elevated transpiration rates (Sack et al. 2016). In addition, a high VPD means low relative humidity, thus low cloud cover and less rain (i.e., a lower CWB). During drought conditions, low water availability at the root level may induce hydraulic dysfunction, impacting meristematic cells, which are necessary for growth (McDowell et al. 2022). Additionally, stomata closure as a response to increased heat stress may limit sap transport from the canopy to the stem and, implicitly, the sugar supply (Adams et al. 2017). Within this framework, drought, associated with or resulting from heat stress, emerges as a key driver of reduced photosynthetic capacity and increased tree mortality (Sevanto et al. 2014). These physiological responses highlight the vulnerability of oaks to specific climate factors, reinforcing predictions that under different climate scenarios, the range of oak may contract in Southern Europe, as suggested by the SDM (Dyderski et al. 2025).

### 4.2 Higher but Persistent Growth Coherence for Oaks From the Potential Range Contraction Area

The higher synchrony values observed for sites projected to experience a range contraction under different climate scenarios, compared to those with potential range persistence, suggest notable differences between the two groups. Over the last few decades, we have observed stable *rbar* values in the expected contraction area, suggesting that these trees have not yet experienced significant changes in stress levels. According to Tejedor et al. (2020), *rbar* values between 0.50 and 0.59 indicate high synchronicity levels, which aligns with our observations in the expected contraction area. This constant within‐population synchrony, combined with the greater climate sensitivity detected in these sites, suggests that a dominant climatic factor strongly and consistently drives oak growth—likely water availability (CWB). Such sustained climatic forcing over time implies that these forests have long been exposed to increased drought‐related stress, and with increasing drought frequency, their risk of experiencing growth decline may intensify. Conversely, *rbar* values from the persistence area (ranging from 0.44 to 0.51) indicate moderate synchrony. The slight variation observed in *rbar* values (0.05, ranging from 0.50 to 0.55) might be associated with the lower climate sensitivity observed for this SDM projection (Figure 3c). Overall, the greater climate sensitivity of oaks from the areas projected to face range contraction is linked to higher growth synchrony, as previously reported by Tejedor et al. (2020).

As hypothesized (H2), our findings reveal higher regional synchrony values (â_C_) in the contraction group, with relatively minor fluctuations between the early and late periods. By contrast, the persistence area exhibited lower overall synchrony levels but demonstrated a more pronounced increase in synchrony from the early to late periods. One of the limitations of this approach may be related to the shorter distances between the plots in the expected contraction area (~500 km, on average) compared to the longer distances in the persistence area (~650 km, on average). Nevertheless, our results suggest distinct responses to environmental constraints in the two SDM regions, which may have important implications for forest dynamics under a changing climate. Furthermore, these patterns appear to be linked to differences in climate sensitivity and the timing of oak growth climatic limitations.

### 4.3 Stronger Temporal Shifts in Climate Sensitivity in Sites From the Range Persistence Area

With the intensification of climate change in recent decades, multiple studies have documented shifts in tree sensitivity to climate (Babst et al. 2019; Jevšenak et al. 2024; Šenfeldr et al. 2024; Wilmking et al. 2020). In our network, this phenomenon—known as non‐stationarity or TSCS (Popa, Jevšenak, et al. 2024)—has been identified for all analyzed climatic factors, albeit with varying intensities, depending on the SDM projections. In terms of the CWB, we observed a similar pattern across the entire network, with no difference between the areas derived from different SDM projections. Specifically, when considering all correlations, regardless of significance, we noted a slight increase in the strength of the correlations (Figure 3c). However, when focusing on the periods with the strongest correlations, a notable increase in the sensitivity of oak to the CWB emerged in the contraction area (Figure 4). These findings are in accordance with the global trend of increasing sensitivity to water availability (Babst et al. 2019). Local studies have reported similar findings for oaks across Europe (Friedrichs et al. 2009; Gribbe et al. 2024; Kasper et al. 2022; Nechita et al. 2019; Scharnweber et al. 2019), supporting the projections of oak persistence in the central and northern parts of Europe, where water availability constraints have not yet intensified. Conversely, in the southern parts of the network, the rising constraints on water availability over recent decades, combined with the predicted increase in the intensity and frequency of droughts, highlight the increasing vulnerability of oak‐dominated forest ecosystems under climate change.

Oak growth is generally negatively correlated with the temperature of the current growing season, while during winter or the previous growing season, we also observed positive temperature effects (Figure 3a). Changes in climate sensitivity, expressed as *Δr* (Figure 3c), indicate stronger changes in areas projected to persist under different climate change scenarios. Most of these changes were linked to the dormant season, where positive correlations have strengthened over time. This can be explained by recent mild winters having favored oak growth in the central and northern parts of the oak distribution in Europe. However, when assessing the temporal shift in oak temperature sensitivity, the optimal model (Figure 4) did not include SDM projections but indicated an overall significant decrease from the early to late periods, suggesting a consistent pattern across the study area. This indicates that SDMs may better reflect conditions important for reproduction and early life stages, consistent with ecological niche theory on which the SDM models are based and with the specificity of species distribution predictors, which are most often bioclimatic variables that limit the survival of individuals (Elith and Leathwick 2009; Peterson et al. 2012). It is important to note, however, that conditions optimal for flowering, seed production, germination, and early seedling growth may differ from those favorable for the growth of mature trees (Hanley et al. 2019; Jastrzębowski and Ukalska 2019). Thus, while SDMs inform us about species' ability to persist under changing environmental conditions, dendrochronological approaches allow the assessment of changes in the quality of the growth conditions of mature trees. Hence, the two research approaches need not be contradictory, and in combination can be complementary, providing valuable results for planning sustainable forest management that takes into account both species persistence and wood production.

We observed a similar, but more pronounced, oak response to the VPD compared to temperature, largely because rising temperatures lead to an exponential increase in VPD (Grossiord et al. 2020). At the European scale, particularly in Central and Eastern Europe, the VPD has shown a continuous increase since the 1980s, although at a slower rate than in the Mediterranean region (Grossiord et al. 2020). When analyzing the correlations across the early and later periods in relation to the three defined seasons, we observed only slight changes in the negative correlation between VPD in the current growing season and oak growth, and a diverse effect for the dormancy period in the persistence area and the previous seasons in the potential contraction area (Figure S10). These findings, combined with the results from linear mixed‐effects models (Figure 4) that indicate a substantial oak sensitivity to VPD, suggest that atmospheric dryness has only a limited impact on oak growth in Europe. However, these findings probably depend on the temporal span of the analysis. When assessing sub‐daily growth from dendrometers, the effect of VPD is often crucial (i.e., the VPD mostly drives daily growth cycles) (Tumajer et al. 2022), but when correlating annual TRWs with monthly VPD values, the influence may be weaker, compared with results of research that use finer‐scale VPD data.

In addition, these results may have been affected by water‐use efficiency and stomatal closure during a period of stress conditions that may differ from those of mesic to xeric sites (Madrigal‐González et al. 2017). Nevertheless, significant differences in drought responses have been reported from oak populations, with some sites being highly sensitive to water availability, while others show little to no sensitivity (Bose et al. 2021; Friedrichs et al. 2009). In particular, at non‐sensitive sites, oaks have struggled to fully recover after drought events. While increased climate sensitivity has been observed in the contraction areas, this does not, on its own, necessarily indicate an imminent decline. However, our findings, particularly stable growth synchrony at the tree level, need to be associated with growth trend analysis to assess the potential for future declines with higher certainty. For another perspective, these sites may already be under prolonged stress, potentially fostering greater plasticity and resilience in response to environmental changes. Additionally, at the southeastern range edge, oak often occurs in specific microhabitats (e.g., in ravines, along rivers, in local depressions) that provide at least partial buffering against water stress. These refugia can effectively delay the impacts of rising temperature or VPD, even if the region is arid or semiarid. However, the loss of climatic suitability at these sites is predicted to occur in the coming decades (based on SDMs), and therefore, the impacts may not be manifested yet.

### 4.4 Novelty and Applicability of the Current Theoretical Framework, and Future and Wider Perspectives

Our findings confirm that, while SDMs are useful for determining the stability of forest ecosystems, they do not provide reliable indications of the growth performance of mature stands. Additionally, our theoretical framework, which relies on dendroecological indicators to assess the vulnerability of oak in the context of climate change and relative to SDM projections, was aimed at building upon previous efforts to combine SDMs with ecophysiological traits (e.g., Benito Garzón et al. 2019; Fréjaville et al. 2019; Gárate‐Escamilla et al. 2019; Rehfeldt et al. 2018). The ability of trees to adapt to changing environmental conditions and maintain populations in specific areas is driven by local adaptation or phenotypic plasticity (Valladares et al. 2014). Phenotypic plasticity – manifested in traits such as the timing of leaf unfolding, leaf senescence, bud burst, seed maturation, or flowering—is strongly influenced by temperature fluctuations and serves as one of the primary adaptive mechanisms of trees (Duputié et al. 2015). By contrast, traits such as height or radial growth tend to respond more slowly to climate variations compared to phenological traits (Benito Garzón et al. 2019; Duputié et al. 2015). With growth‐related adaptations occurring over relatively long timescales, tree‐ring data offer valuable information for assessing these patterns over decades to centuries. This underscores the importance of incorporating dendroecological indicators into SDMs alongside other functional and ecophysiological indicators. Integrating dendrochronology with ecological and physiological research may enhance our understanding of forest responses to climate change, which is essential for developing adaptive forest management strategies that promote the long‐term sustainability of forest ecosystems.

Modeling tree growth as a function of climate, and identifying specific thresholds at which growth shifts from energy limitation to water limitation, particularly in relation to oak distribution in Central and Eastern Europe, represents a future research direction, focusing more on the impacts of climate change on the productivity function of forests (Klesse et al. 2024). Furthermore, compiling a pan‐European network of recent tree‐ring data that integrates information from the western and southern parts of Europe may offer an integrated view of the species' adaptability and plasticity of oak along larger spatial and climatic gradients, as shown for other species (Martinez Del Castillo et al. 2024).

One limitation of this study was the potential non‐linearity in climate–growth relationships, which was not explicitly accounted for and may have influenced our findings (Leifsson et al. 2024; Wilmking et al. 2020). Additionally, the models do not account for interaction effects between different climate factors. Such interactions can substantially influence tree growth responses, as the effect of one climatic variable may depend on the level of another. For instance, previous research has demonstrated that trees exhibit a higher sensitivity to specific climatic factors when these factors either limit growth (e.g., water availability) or exceed physiological tolerance thresholds (e.g., temperature, VPD) (Jiang et al. 2024). Given the complexity of climate change and the interplay of these limiting or inhibiting factors, our large‐scale and temporal assessment may not have fully captured all the nuances of oak climate sensitivity. To improve our understanding of this species' adaptive capacity, further research is needed in the direction of non‐linearity or interaction effects in climate–growth relationships.

In conclusion, our analyses revealed that, in the contraction area projected by the SDM, oaks exhibit increased sensitivity to water availability and show negative relationships with temperature and VPD during the growing seasons. These interdependencies have changed only slightly over the last few decades. Furthermore, tree growth synchrony has remained stable over the last seven decades, suggesting that, from a growth perspective, the potential range contraction projected by the SDM does not yet fully align with the dendroecological indicators. In the northern sites (i.e., the persistence area projected by the SDM), we observed stronger temporal shifts in climate sensitivity, especially for temperature and VPD, and variable growth synchrony. Ultimately, these findings suggest that *Q. robur* has not yet been severely affected by climate change over the last few decades, and that, in persistence areas (i.e., Central and Northern Europe) the species copes better than in contraction areas (i.e., Southern and Eastern Europe).

## Author Contributions

**Andrei Popa:** conceptualization, data curation, formal analysis, investigation, methodology, resources, software, validation, visualization, writing – original draft, writing – review and editing. **Jernej Jevšenak:** conceptualization, investigation, methodology, supervision, validation, writing – review and editing. **Marcin Dyderski:** conceptualization, formal analysis, methodology, resources, software, validation, writing – review and editing. **Radosław Puchałka:** conceptualization, methodology, resources, writing – review and editing. **Allan Buras:** methodology, validation, writing – review and editing. **Ionel Popa:** conceptualization, funding acquisition, project administration, resources, supervision, writing – review and editing. **Martin Wilmking:** resources, writing – review and editing. **Aleksandra Kalisty:** resources, writing – review and editing. **Catalin‐Constantin Roibu:** resources, writing – review and editing. **Marcin Jakubowski:** resources, writing – review and editing. **Eric Thurm:** resources, writing – review and editing. **Martin Šenfeldr:** resources, writing – review and editing. **Marko Smiljanić:** resources, writing – review and editing. **Ernst van der Maaten:** resources, writing – review and editing. **Jan Esper:** resources, writing – review and editing. **Edurne Martinez del Castillo:** resources, writing – review and editing. **Vaclav Treml:** resources, writing – review and editing. **Jan Tumajer:** resources, writing – review and editing. **Tzvetan Zlatanov:** resources, writing – review and editing. **Roberts Matisons:** resources, writing – review and editing. **Gheorghe Florenta:** resources, writing – review and editing. **Veronica Florenta:** resources, writing – review and editing. **Maksym Netsvetov:** resources, writing – review and editing. **Vladislav Grati:** resources, writing – review and editing. **Andreas Burger:** resources, writing – review and editing. **Karolina Janecka:** resources, writing – review and editing. **Saša Kostić:** resources, writing – review and editing. **Kamil Pilch:** resources, writing – review and editing. **Diāna Jansone:** resources, writing – review and editing. **Agnese Liepiņa:** resources, writing – review and editing. **Yulia Prokopuk:** resources, writing – review and editing. **Oleksandr Sylenko:** resources, writing – review and editing. **Mátyás Árvai:** resources, writing – review and editing. **Achim Bräuning:** resources, writing – review and editing. **Cristina Marques:** resources, writing – review and editing. **Martin Häusser:** resources, writing – review and editing. **Emil Horváth:** resources, writing – review and editing. **Jakub Jeleń:** resources, writing – review and editing. **Ryszard Kaczka:** resources, writing – review and editing. **Zoltán Kern:** resources, writing – review and editing. **Tomáš Kolář:** resources, writing – review and editing. **Marcin Koprowski:** resources, writing – review and editing. **Sandra Metslaid:** resources, writing – review and editing. **András Morgós:** resources, writing – review and editing. **Oleksandr Khodosovtsev:** resources, writing – review and editing. **Aleksei Potapov:** resources, writing – review and editing. **Michal Rybníček:** resources, writing – review and editing. **Irena Sochová:** resources, writing – review and editing. **Kristina Sohar:** resources, writing – review and editing. **Vasyl Budzhak:** resources, writing – review and editing. **Ewa Zin:** resources, writing – review and editing. **Tassilo Schneider:** resources, writing – review and editing. **Wojciech Gil:** resources, writing – review and editing. **Marcin Klisz:** conceptualization, funding acquisition, methodology, project administration, resources, supervision, validation, writing – review and editing.

## Conflicts of Interest

The authors declare no conflicts of interest.

## Supporting information


**Data S1:** gcb70567‐sup‐0001‐supinfo.docx.

## Data Availability

The data supporting the findings of this study are available at https://doi.org/10.5281/zenodo.16894677. The E‐OBS climate data used in this study can be accessed at http://surfobs.climate.copernicus.eu. The climatic bioindicators can be accessed at https://www.doi.org/10.16904/envidat.332. Species distribution models projections can be accessed at https://doi.org/10.6084/m9.figshare.24139722.
