# Supplementary material for: Spatiotemporal Variability of Dendroecological Indicators in Pedunculate Oak (Quercus robur L.) Tree‐Rings Across Europe in Relation to Species Distribution Models
Source: Glob Chang Biol. 2025 Oct 30;31(11):e70567. doi: 10.1111/gcb.70567 (PMC12573224; doi:10.1111/gcb.70567)
Supplement: Supplementary file 1 — Data S1: gcb70567‐sup‐0001‐supinfo.docx. [file GCB-31-e70567-s001.docx]

**Spatiotemporal variability of dendroecological indicators in pedunculate oak (*Quercus robur* L.) tree-rings across Europe in relation to species distribution models**

Andrei Popa^1,2,*^, Jernej Jevšenak^3^, Marcin Dyderski^4^, Radosław Puchałka^5,6^, Allan Buras^7^, Ionel Popa^1,8^, Martin Wilmking^9^, Aleksandra Kalisty^10^, Catalin-Constantin Roibu^11^, Marcin Jakubowski^12^, Eric Thurm^13^, Martin Šenfeldr^14^, Marko Smiljanić^9^, Ernst van der Maaten^15^, Jan Esper^16,17^, Edurne Martinez del Castillo^16^, Vaclav Treml^18^, Jan Tumajer^18^, Tzvetan Zlatanov^19^, Roberts Matisons^20^, Gheorghe Florenta^21,22^, Veronica Florenta^22^, Maksym Netsvetov^23,24^, Vladislav Grati^11,22^, Andreas Burger^9^, Karolina Janecka^25^, Saša Kostić^26^, Kamil Pilch^27^, Diāna Jansone^20^, Agnese Liepiņa^20^, Yulia Prokopuk^23,24^, Oleksandr Sylenko^23^, Mátyás Árvai^28^, Achim Bräuning^29^, Cristina Marques^30^, Martin Häusser^29^, Emil Horváth^31^, Jakub Jeleń^32^, Ryszard Kaczka^17^, Zoltán Kern^33,34^, Tomáš Kolář^35,36^, Marcin Koprowski^5,6^, Sandra Metslaid^30^, András Morgós^37^, Oleksandr Khodosovtsev^38^, Aleksei Potapov^30^, Michal Rybníček^35,36^, Irena Sochová^35,36^, Kristina Sohar^39^, Vasyl Budzhak^23,40^, Ewa Zin^27,41^, Tassilo Schneider^9^, Wojciech Gil^42^, Marcin Klisz^43^

*corresponding author ([popa.andrei.dorna@gmail.com](mailto:popa.andrei.dorna@gmail.com))

^1^National Institute for Research and Development in Forestry “Marin Drăcea”, Bucharest, Romania

^2^Faculty of Silviculture and Forest Engineering, Transilvania University of Brasov, Brasov, Romania

^3^Department for Forest and Landscape Planning and Monitoring, Slovenian Forestry Institute, Ljubljana, Slovenia

^4^Institute of Dendrology, Polish Academy of Sciences, Poznan, Poland

^5^Department of Ecology and Biogeography, Faculty of Biological and Veterinary Sciences, Nicolaus Copernicus University, Toruń, Poland,

^6^Centre for Climate Change Research, Nicolaus Copernicus University, Toruń, Poland

^7^Land Surface-Atmosphere Interactions, TU Munich, Freising, Germany

^8^Center for Mountain Economy, Vatra Dornei, Romania

^9^Institute for Botany and Landscape Ecology, University of Greifswald, Greifswald, Germany

^10^Forsite Consultants Ltd, Prince Albert, Canada

^11^Forest Biometrics Laboratory, Faculty of Forestry, "Stefan cel Mare" University of Suceava, Romania

^12^Faculty of Forestry and Wood Technology, Poznań University of Life Sciences, Poland;

^13^Landesforstanstalt Mecklenburg-Vorpommern, Department of Forest Planning/Forest Research/Information Systems, Research Unit Silviculture and Forest Growth, Schwerin, Germany

^14^Department of Forest Botany, Dendrology and Geobiocoenology, Faculty of Forestry and Wood Technology, Mendel University in Brno, Brno, Czech Republic

^15^Chair of Forest Growth and Woody Biomass Production, TU Dresden, Germany

^16^Department of Geography, Johannes Gutenberg University, Mainz, Germany

^17^Global Change Research Institute of the Czech Academy of Sciences (Czech- Globe), Brno, Czech Republic

^18^Charles University, Faculty of Science, Department of Physical Geography and Geoecology, Prague, Czech Republic

^19^Institute of Biodiversity and Ecosystem Research, Bulgarian Academy of Sciences

^20^Latvian State Forest Research Institute "Silava", Salaspils, Latvia

^21^Forest Research and Management Institute, Chisinau, Moldova;

^22^Moldova State University, Chisinau, Moldova

^23^Institute for Evolutionary Ecology of the National Academy of Sciences of Ukraine, Kyiv, Ukraine

^24^BIOGECO, INRAE, University of Bordeaux, Cestas, France

^25^University of Geneva, Institute for Environmental Sciences, Switzerland

^26^Institute of Lowland Forestry and Environment, University of Novi Sad, Serbia

^27^Dendrolab IBL, Department of Natural Forests, Forest Research Institute (IBL), Białowieża, Poland

^28^Institute for Soil Sciences, HUN-REN Centre for Agricultural Research, Budapest, Hungary

^29^Institute of Geography, Friedrich-Alexander-Universität Erlangen-Nürnberg

^30^Chair of Forest and Land Management and Wood Processing Technologies, Estonian University of Life Sciences, Estonia

^31^independent researcher, Sárkeresztes, Hungary

^32^Department of Forest Management, Dendrometry and Economics of Forestry, Warsaw University of Life Sciences, Poland

^33^Institute for Geological and Geochemical Research, HUN-REN Research Centre for Astronomy and Earth Sciences, Budapest, Hungary

34CSFK, MTA Centre of Excellence, Budapest, Hungary

^35^Faculty of Forestry and Wood Technology, Mendel University in Brno, Brno, Czech Republic

^36^Global Change Research Institute of the Czech Academy of Sciences, Bělidla 4a, 603 00 Brno, Czech Republic

^37^Consart Bt., Budapest, Hungary

^38^Kherson State University, Ivano-Frankivsk, Ukraine; M.G. Kholodny Institute of Botany, Kyiv, Ukraine

^39^Department of Geography, Institute of Ecology and Earth Sciences, University of Tartu, Tartu, Estonia

^40^Falz-Fein Biosphere Reserve “Askania Nova”, Ukraine

^41^Southern Swedish Forest Research Centre, Swedish University of Agricultural Sciences (SLU), Alnarp, Sweden

^42^Department of Silviculture and Genetics, Forest Research Institute (IBL), Sękocin Stary, Poland

^43^Dendrolab IBL, Department of Silviculture and Genetics, Forest Research Institute (IBL), Sękocin Stary, Poland

**Supplementary Material**


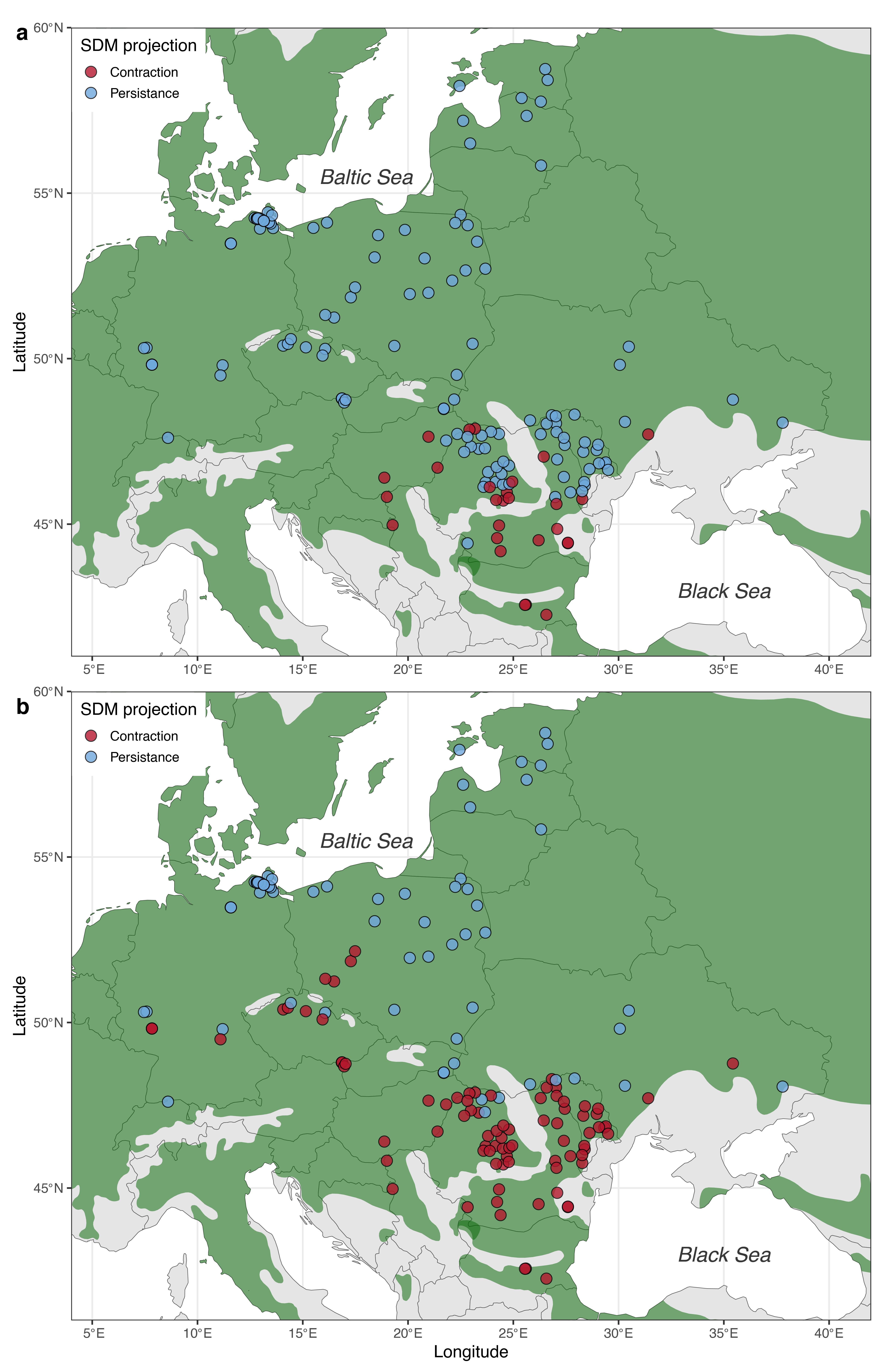


Figure S1 Map of the study area and points indicating study site locations; colors indicating the species distribution model (SDM) projection under the climate scenario SSP245 (**a**) and under the climate scenario SSP585 (**b**). The shaded green area depicts the distribution of *Quercus robur* L. in Europe (Caudullo et al., 2017).


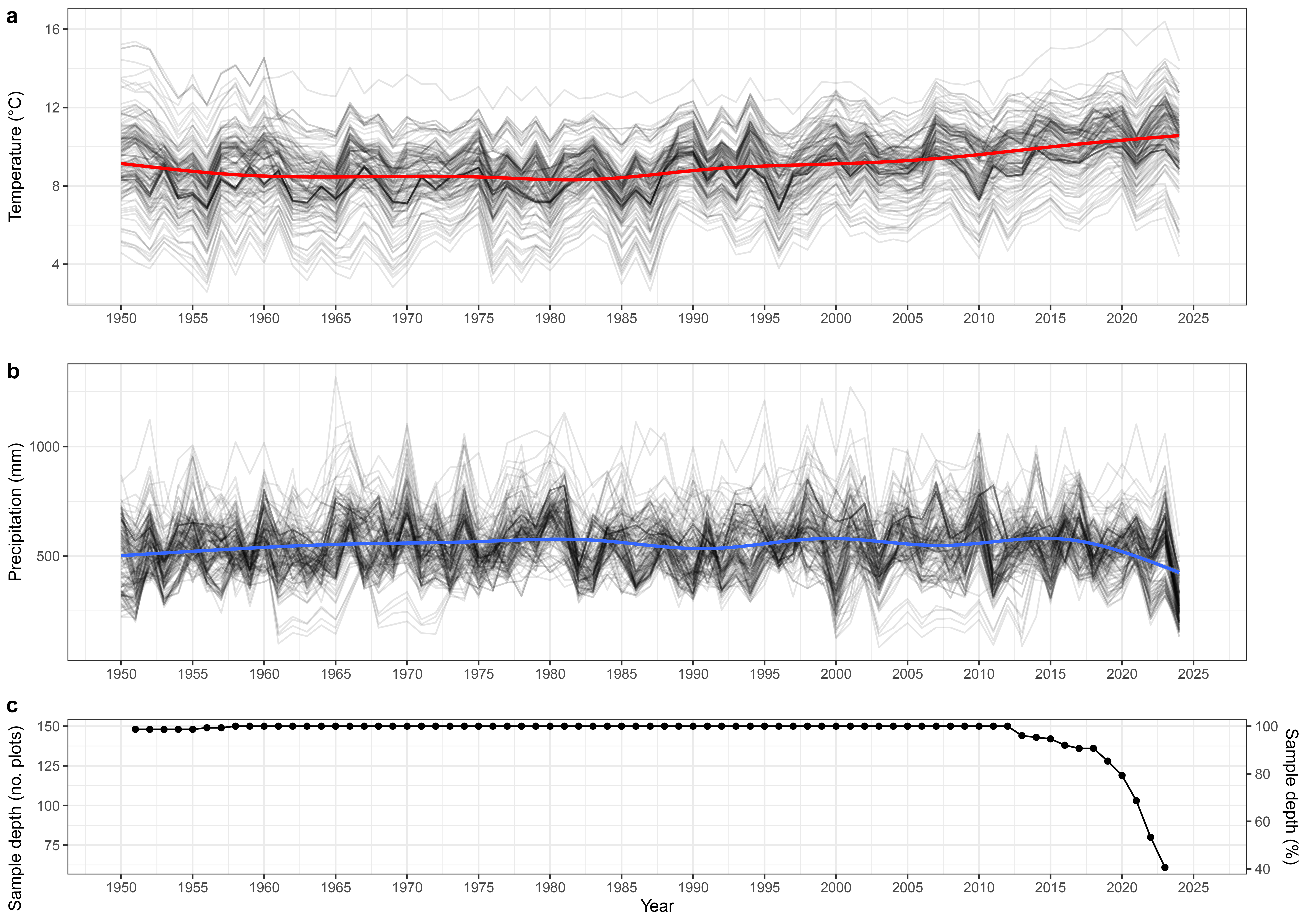


Figure S2 (**a**) Mean annual temperature at the plot level (black lines) and the overall trend since 1950 (red). (**b**) Annual precipitation at the plot level (black lines) and the overall trend since 1950 (blue). (**c**) Number of chronologies that cover the study period


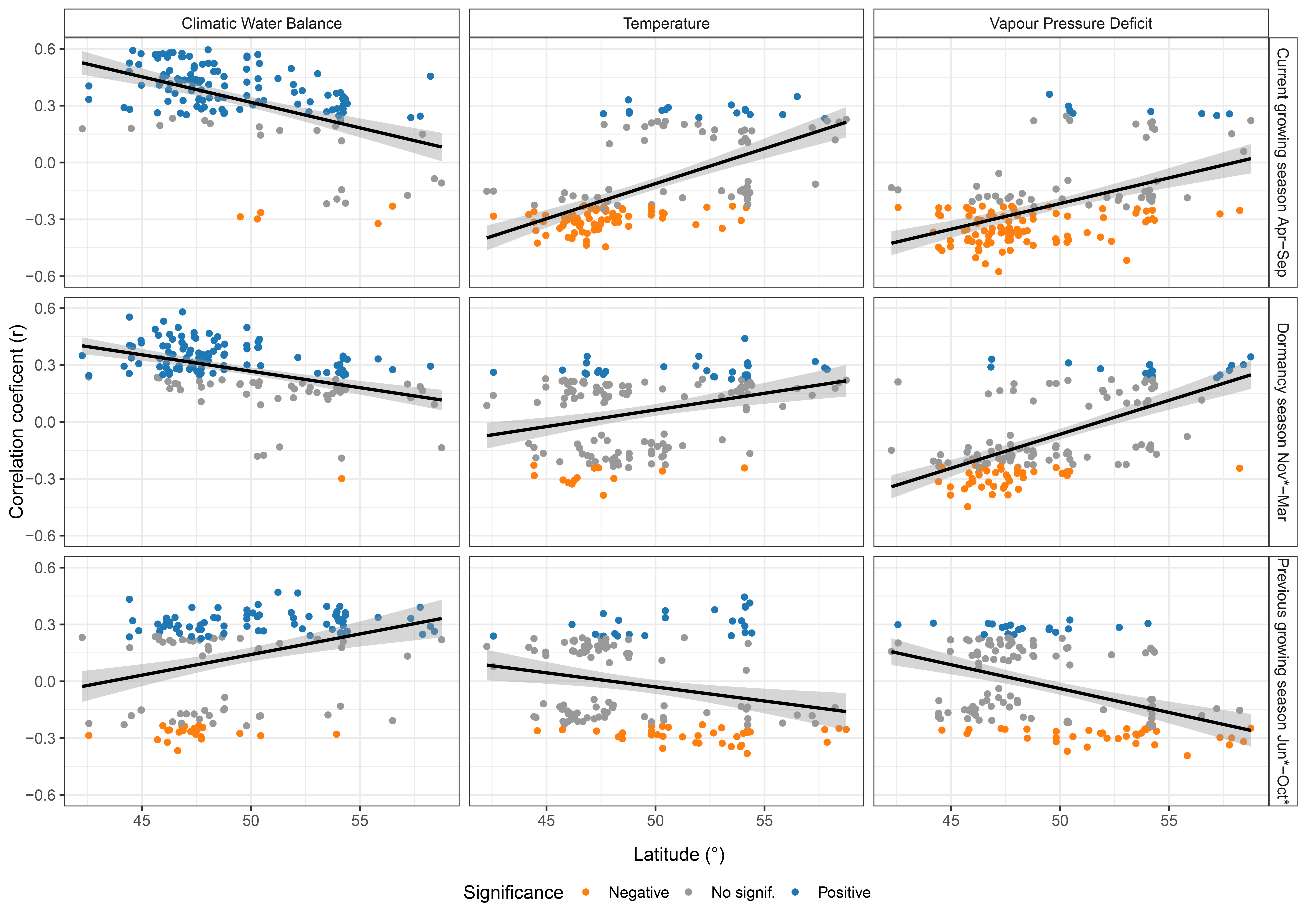


Figure S3 The geographic variability of oak’s sensitivity to climate, the regressions of correlation coefficients ~ latitude. Abbreviated name of month with or without asterisk refers to the month in the year preceding and current year of ring formation, respectively.


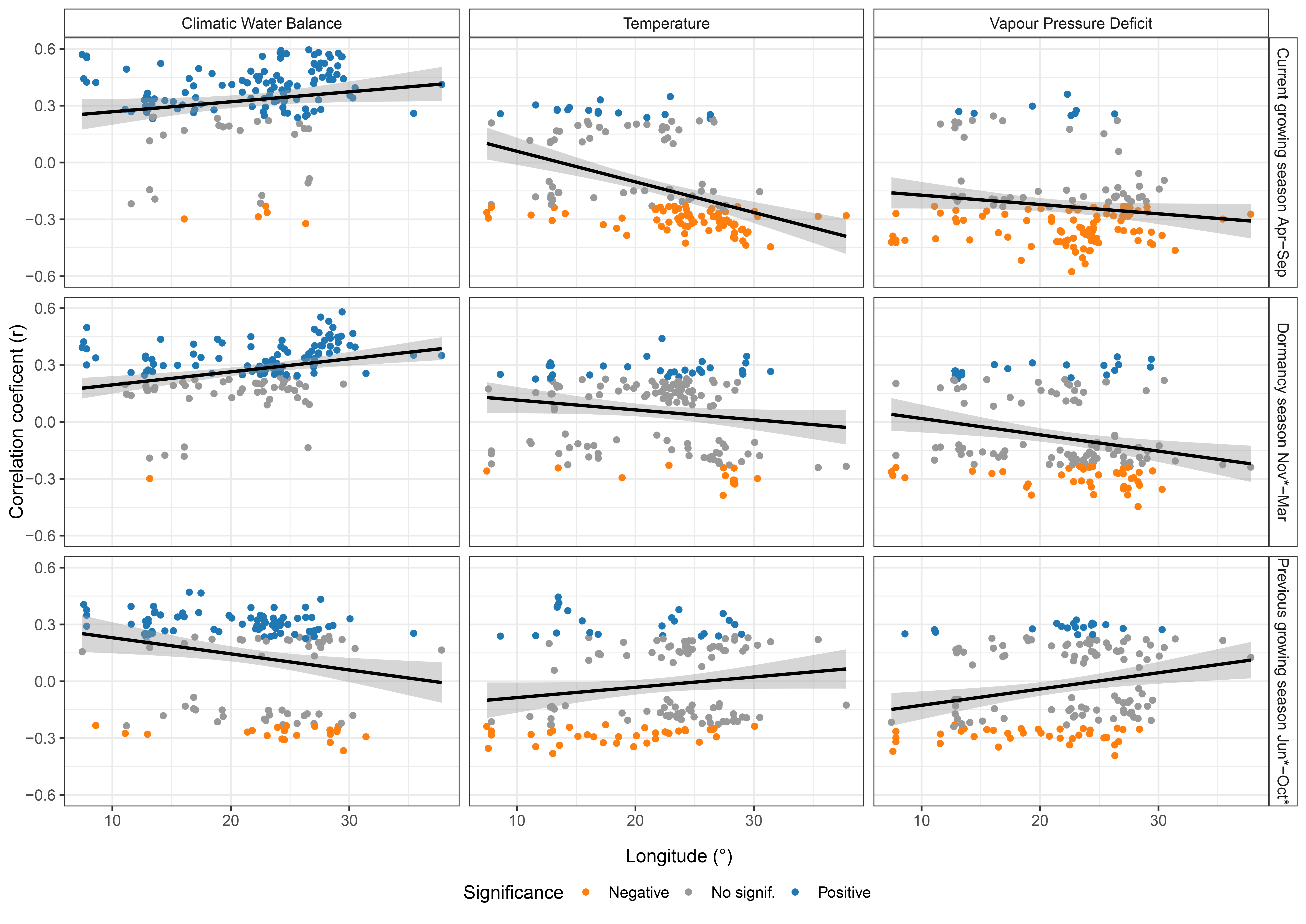


Figure S4 The geographic variability of oak’s sensitivity to climate, the regressions of correlation coefficients ~ longitude. Abbreviated name of month with or without asterisk refers to the month in the year preceding and current year of ring formation, respectively.


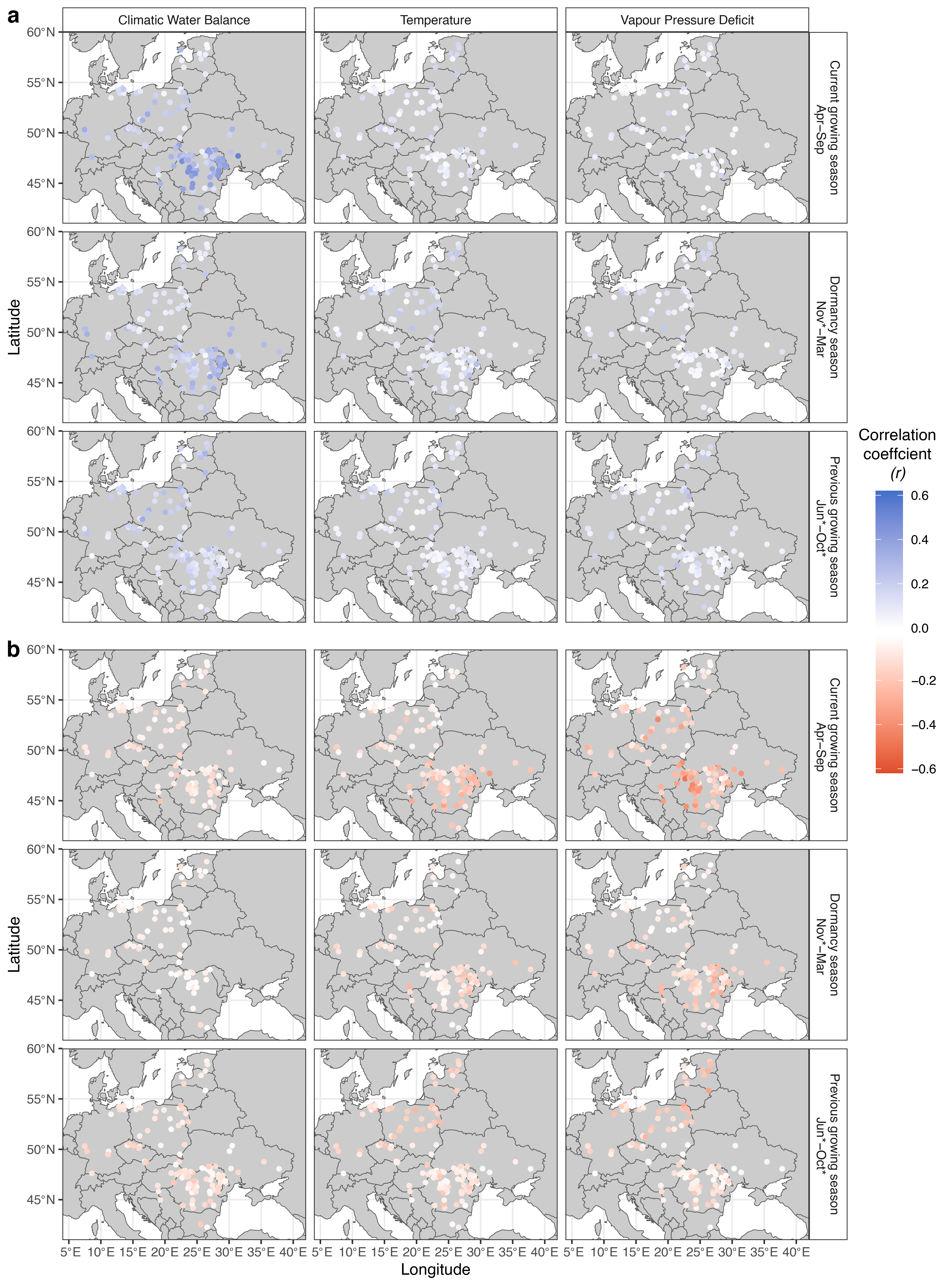


Figure S5 Median correlation of all seasons regardless of significance. (**a**) The upper 3 rows show the median of only positive correlations in relation to climate parameters and seasons. (**b**) The lower 3 rows show the median of only negative correlations in relation to climate parameters and seasons. Abbreviated name of month with or without asterisk refers to the month in the year preceding and current year of ring formation, respectively.


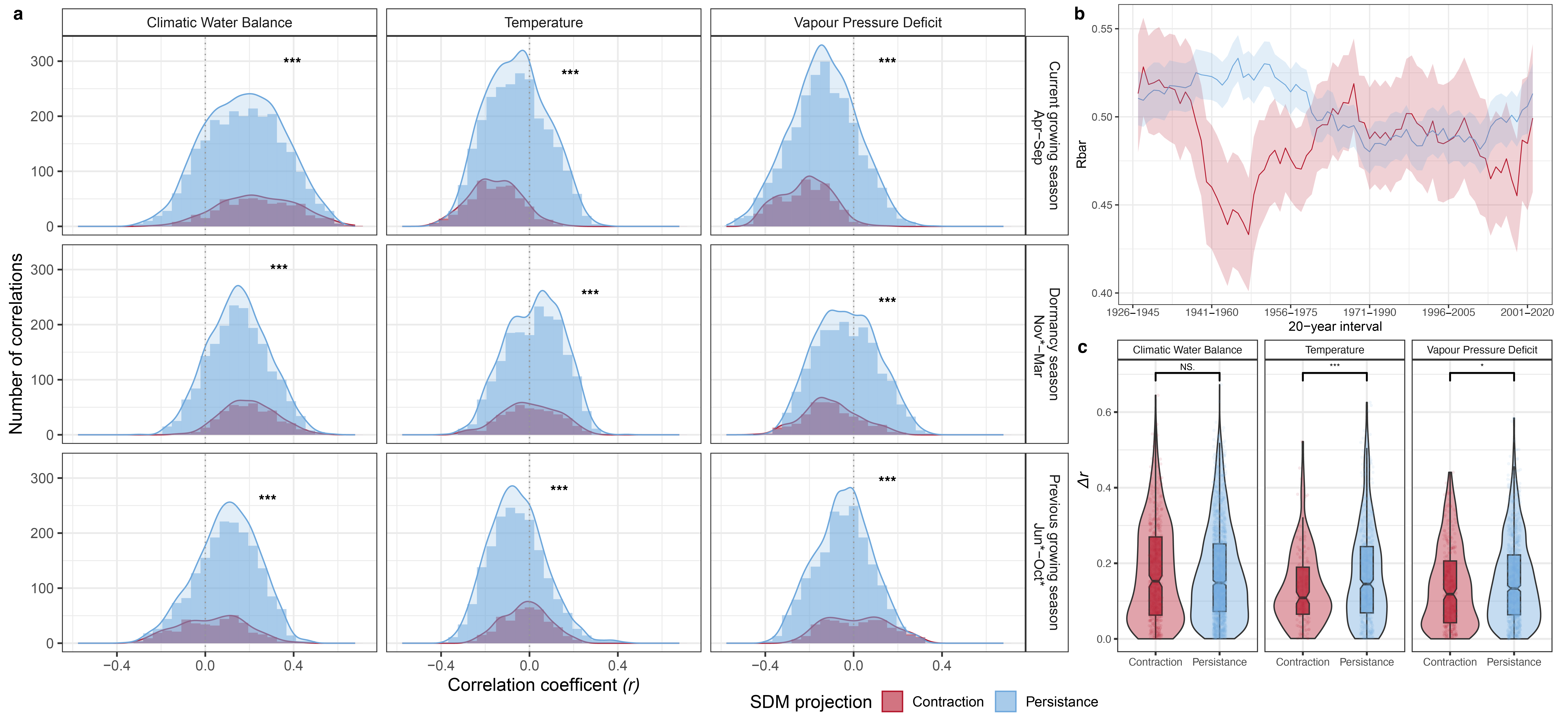


Figure S6 (**a**) Distributions of the correlation coefficients in areas of contraction and persistence across different seasons based on SDM under the SSP245 scenario. (**b**) Mean growth synchrony (rbar) within the SDM projections. Shaded areas represent the standard error; the x-axis presents the interval for which the *rbar* was calculated, indicated by the end of the reference window. (**c**) The temporal shift of climate sensitivity, where *𝚫r* depicts the absolute difference in the correlations between the late and early periods, respectively. In panel **a** abbreviated name of month with or without asterisk refers to the month in the year preceding and current year of ring formation, respectively.


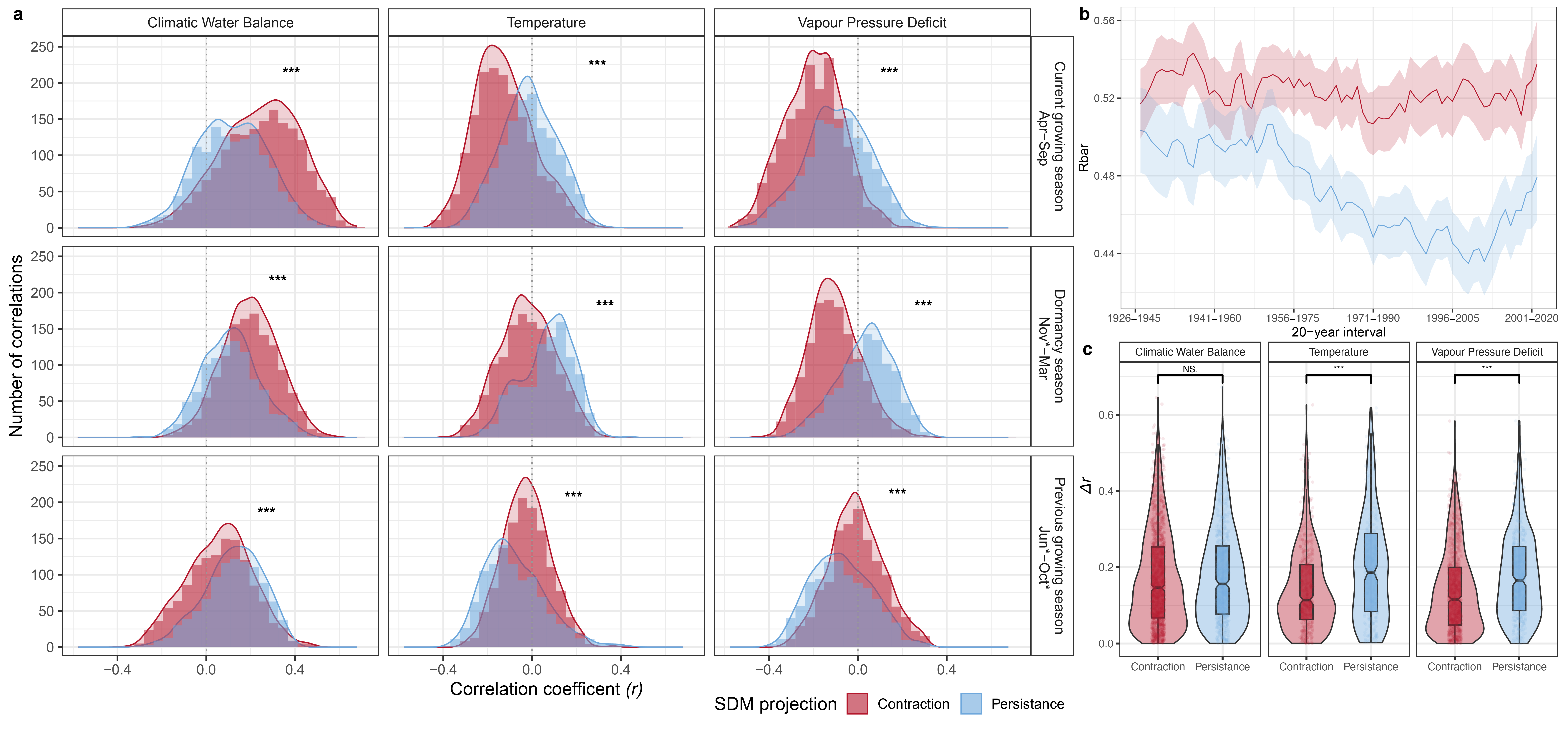


Figure S7 (**a**) Distributions of the correlation coefficients in areas of contraction and persistence across different seasons based on SDM under the SSP585 scenario. (**b**) Mean growth synchrony (rbar) within the SDM projections. Shaded areas represent the standard error; the x-axis presents the interval for which the *rbar* was calculated, indicated by the end of the reference window. (**c**) The temporal shift of climate sensitivity, where *𝚫r* depicts the absolute difference in the correlations between the late and early periods, respectively. In panel **a** abbreviated name of month with or without asterisk refers to the month in the year preceding and current year of ring formation, respectively.

Table S1 Summary of the fitted mixed-effects models for the sensitivity of oak to climatic water balance (CWB), mean temperature, and vapor pressure deficit (VPD).

|  | CWB | | | | | Temperature | | | | | VPD | | | | |
| --- | --- | --- | --- | --- | --- | --- | --- | --- | --- | --- | --- | --- | --- | --- | --- |
| *Fixed effects* | $\beta$ | *CI* | *t* | *p* | $\beta$ | | *CI* | *t* | *p* | $\beta$ | | *CI* | *t* | *p* |  |
| (Intercept) | 0.052 | -0.155, 0.258 | 0.488 | 0.625 | 0.220 | | 0.032, 0.408 | 2.297 | **0.022** | 0.062 | | -0.162, 0.287 | 0.543 | 0.587 |  |
| BIO18 | -0.000 | -0.001, -0.000 | -2.574 | **0.010** | 0.000 | | -0.000, 0.001 | 1.753 | 0.080 | 0.000 | | -0.000, 0.001 | 0.917 | 0.359 |  |
| BIO9 | -0.006 | -0.011, -0.001 | -2.242 | **0.025** | 0.004 | | -0.001, 0.008 | 1.630 | 0.103 | 0.000 | | -0.009, 0.010 | 0.075 | 0.940 |  |
| BIO10 | 0.011 | 0.003, 0.019 | 2.555 | **0.011** | -0.019 | | -0.027, -0.011 | -4.937 | **<0.001** | 0.005 | | -0.001, 0.010 | 1.658 | 0.097 |  |
| BIO11 | -0.002 | -0.011, 0.007 | -0.359 | 0.719 | 0.001 | | -0.007, 0.009 | 0.296 | 0.767 | -0.012 | | -0.021, -0.003 | -2.657 | **0.008** |  |
| Period [late] | 0.022 | 0.013, 0.030 | 4.848 | **<0.001** | 0.047 | | 0.039, 0.055 | 11.607 | **<0.001** | 0.021 | | 0.013, 0.029 | 4.967 | **<0.001** |  |
| SDM projection [persistence] | 0.003 | -0.029, 0.035 | 0.179 | 0.858 | -0.022 | | -0.051, 0.007 | -1.470 | 0.142 | 0.021 | | -0.013, 0.056 | 1.202 | 0.229 |  |
| age | 0.000 | -0.000, 0.000 | 0.006 | 0.995 | 0.000 | | -0.000, 0.000 | 0.689 | 0.491 | 0.000 | | -0.000, 0.000 | 0.409 | 0.682 |  |
| Period [late] × SDM projection [persistence] | -0.032 | -0.044, -0.020 | -5.185 | **<0.001** | 0.018 | | 0.007, 0.029 | 3.216 | **0.001** | 0.002 | | -0.009, 0.013 | 0.344 | 0.730 |  |
| *Random Effects* | | | | | | | | | | | | | | | |
| σ^2^ | 0.035 | | | | | 0.029 | | | | | 0.020 | | | | |
| τ_00_ _cod_ | 0.004 | | | | | 0.003 | | | | | 0.022 | | | | |
| ICC | 0.102 | | | | | 0.101 | | | | | 0.691 | | | | |
| No. corr. / No. plots | 15138 / 150 | | | | | 15138 / 150 | | | | | 15138 / 150 | | | | |
| R^2^_m_ / R^2^_c_ | 0.031 / 0.130 | | | | | 0.062 / 0.156 | | | | | 0.038 / 0.164 | | | | |

One model was fitted individually for each of the climate factors. The response variables are correlation coefficients between RWI and climatic factors. The fixed part of the model included BIO18 (mean monthly precipitation amount of the warmest quarter), BIO9 (mean daily mean air temperatures of the driest quarter), BIO10 (mean daily mean air temperatures of the warmest quarter), BIO11 (mean daily mean air temperatures of the coldest quarter), mean age of the stands (age), periods (early, late), and SDM projection (contraction, persistence) as categorical variables, and interaction between periods and SDM projections. The random part of the model included the site and the season. This model is based on all correlations regardless of significance; as a sensitivity analysis, we present in Table S2 similar models using only significant correlations in early or late periods, and in Table S3, a model in which we used climatic parameters as a fixed factor. Values represent the estimates of regression coefficients (β), 95% confidence intervals (CI), the *t* statistic, and the associated p-value of significance (bold values stand for significant fixed effects, *p* < 0.05). σ^2^ represents the variance of residuals, τ variance caused by random effects, ICC intra-class correlation coefficient R^2^_m_ is the marginal R^2^, and R^2^_c_ is the conditional R^2^. The low marginal R^2^ explained by the fixed effects of the optimized models might be a consequence of data heterogeneity.

Table S2 Summary of the fitted mixed-effects models for the sensitivity of oak to climatic water balance (CWB), mean temperature, and vapor pressure deficit (VPD) using only significant correlations.

|  | CWB | | | | | Temperature | | | | | VPD | | | | |
| --- | --- | --- | --- | --- | --- | --- | --- | --- | --- | --- | --- | --- | --- | --- | --- |
| *Fixed effects* | $\beta$ | *CI* | *t* | *p* | $\beta$ | | *CI* | *t* | *p* | $\beta$ | | *CI* | *t* | *p* |  |
| (Intercept) | 0.333 | 0.218, 0.448 | 5.682 | **<0.001** | 0.706 | | -0.086, 1.499 | 1.749 | 0.081 | 0.087 | | -0.554, 0.727 | 0.265 | 0.791 |  |
| BIO18 | -0.000 | -0.000, 0.000 | -1.441 | 0.150 | 0.001 | | -0.001, 0.002 | 0.958 | 0.338 | 0.001 | | -0.000, 0.002 | 1.392 | 0.164 |  |
| BIO9 | -0.002 | -0.004, 0.001 | -1.109 | 0.268 | 0.009 | | -0.010, 0.029 | 0.918 | 0.359 | 0.029 | | 0.001, 0.057 | 2.014 | **0.044** |  |
| BIO10 | 0.007 | 0.002, 0.011 | 2.856 | **0.004** | -0.057 | | -0.088, -0.025 | -3.513 | **<0.001** | 0.002 | | -0.014, 0.018 | 0.224 | 0.823 |  |
| BIO11 | -0.002 | -0.007, 0.003 | -0.687 | 0.492 | 0.012 | | -0.021, 0.046 | 0.715 | 0.475 | -0.028 | | -0.054, -0.003 | -2.174 | **0.030** |  |
| Period [late] | 0.012 | 0.005, 0.020 | 3.382 | **0.001** | 0.114 | | 0.061, 0.168 | 4.225 | **<0.001** | 0.078 | | 0.052, 0.103 | 5.925 | **<0.001** |  |
| SDM projection [persistence] | -0.003 | -0.021, 0.015 | -0.340 | 0.734 | -0.163 | | -0.290, -0.036 | -2.519 | **0.012** | 0.052 | | -0.048, 0.151 | 1.023 | 0.306 |  |
| age | -0.000 | -0.000, 0.000 | -1.076 | 0.282 | 0.000 | | -0.001, 0.001 | 0.599 | 0.549 | -0.000 | | -0.001, 0.000 | -1.072 | 0.284 |  |
| Period [late] × SDM projection [persistence] | 0.000 | -0.011, 0.012 | 0.082 | 0.935 | 0.180 | | 0.111, 0.249 | 5.119 | **<0.001** | 0.056 | | 0.018, 0.095 | 2.846 | **0.004** |  |
| *Random Effects* | | | | | | | | | | | | | | | |
| σ^2^ | 0.005 | | | | | 0.059 | | | | | 0.034 | | | | |
| τ_00_ _cod_ | 0.001 | | | | | 0.048 | | | | | 0.037 | | | | |
| ICC | 0.157 | | | | | 0.450 | | | | | 0.522 | | | | |
| No. corr. / No. plots | 2900 / 147 | | | | | 1221 / 140 | | | | | 2035 / 145 | | | | |
| R^2^_m_ / R^2^_c_ | 0.047 / 0.197 | | | | | 0.170 / 0.543 | | | | | 0.142 / 0.590 | | | | |

One model was fitted individually for each of the climate factors. The response variables are correlation coefficients between RWI and climatic factors. The fixed part of the model included BIO18 (mean monthly precipitation amount of the warmest quarter), BIO9 (mean daily mean air temperatures of the driest quarter), BIO10 (mean daily mean air temperatures of the warmest quarter), BIO11 (mean daily mean air temperatures of the coldest quarter), mean age of the stands (age), periods (early, late), and SDM projection (contraction, persistence) as categorical variables, and interaction between periods and SDM projections. The random part of the model included the site and the season. Values represent the estimates of regression coefficients (β), 95% confidence intervals (CI), the *t* statistic, and the associated p-value of significance (bold values stand for significant fixed effects, *p* < 0.05). σ^2^ represents the variance of residuals, τ variance caused by random effects, ICC intra-class correlation coefficient R^2^_m_ is the marginal R^2^, and R^2^_c_ is the conditional R^2^. The low marginal R^2^ explained by the fixed effects of the optimized models might be a consequence of data heterogeneity.





Figure S8 Diagnostic plots for the models presented in Table S1 for CWB (a), temperature (b), and VPD (c). Figures were generated using the *check_model()* function from ‘performance’ R package (Lüdecke et al., 2021)


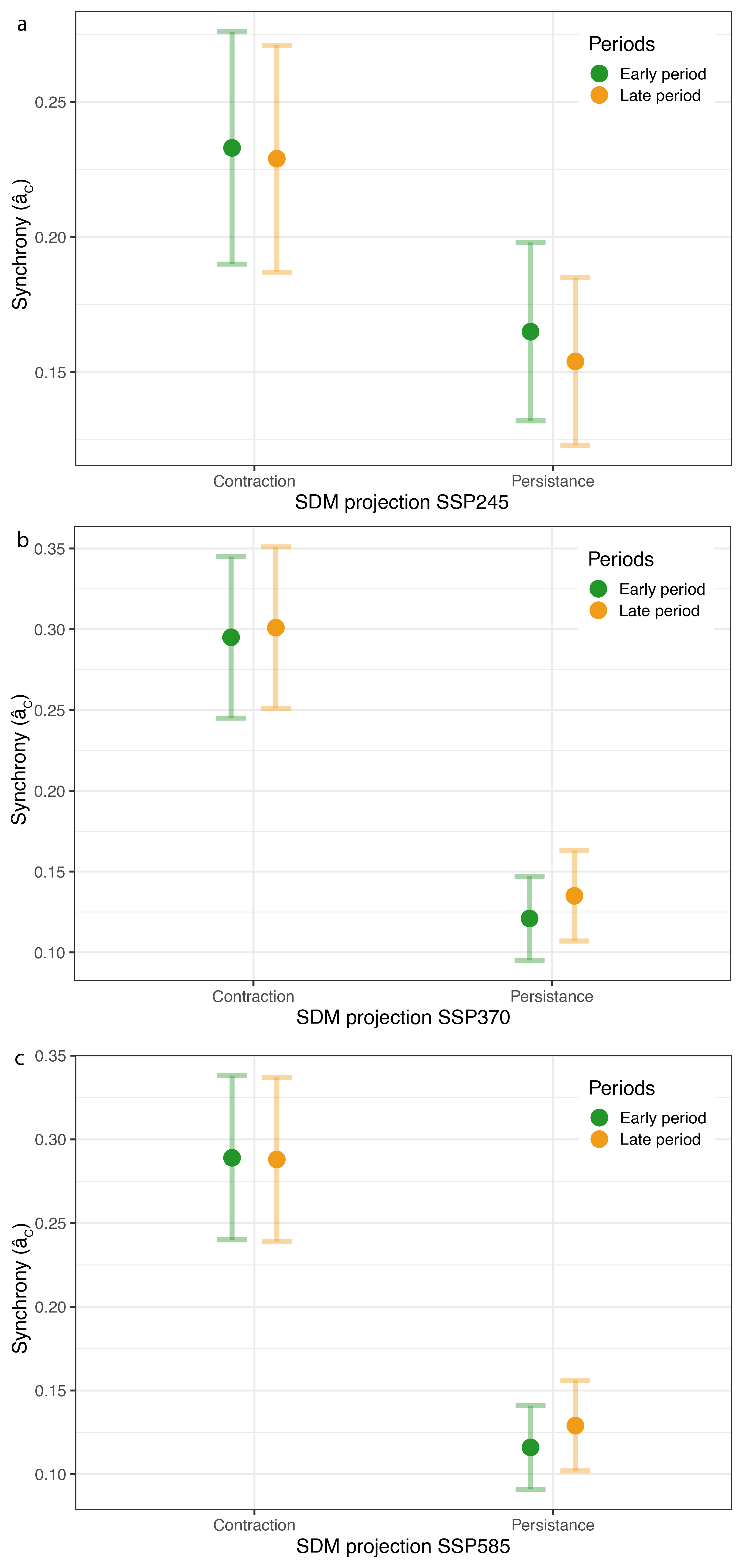


Figure S9 Regional growth synchrony within the SDM projections based on SSP245 scenarios (a), on SSP370 scenarios (b), and on SSP585 (c) using a variance-covariance model, the homoscedastic variant of the full model defined by (Shestakova et al., 2014)


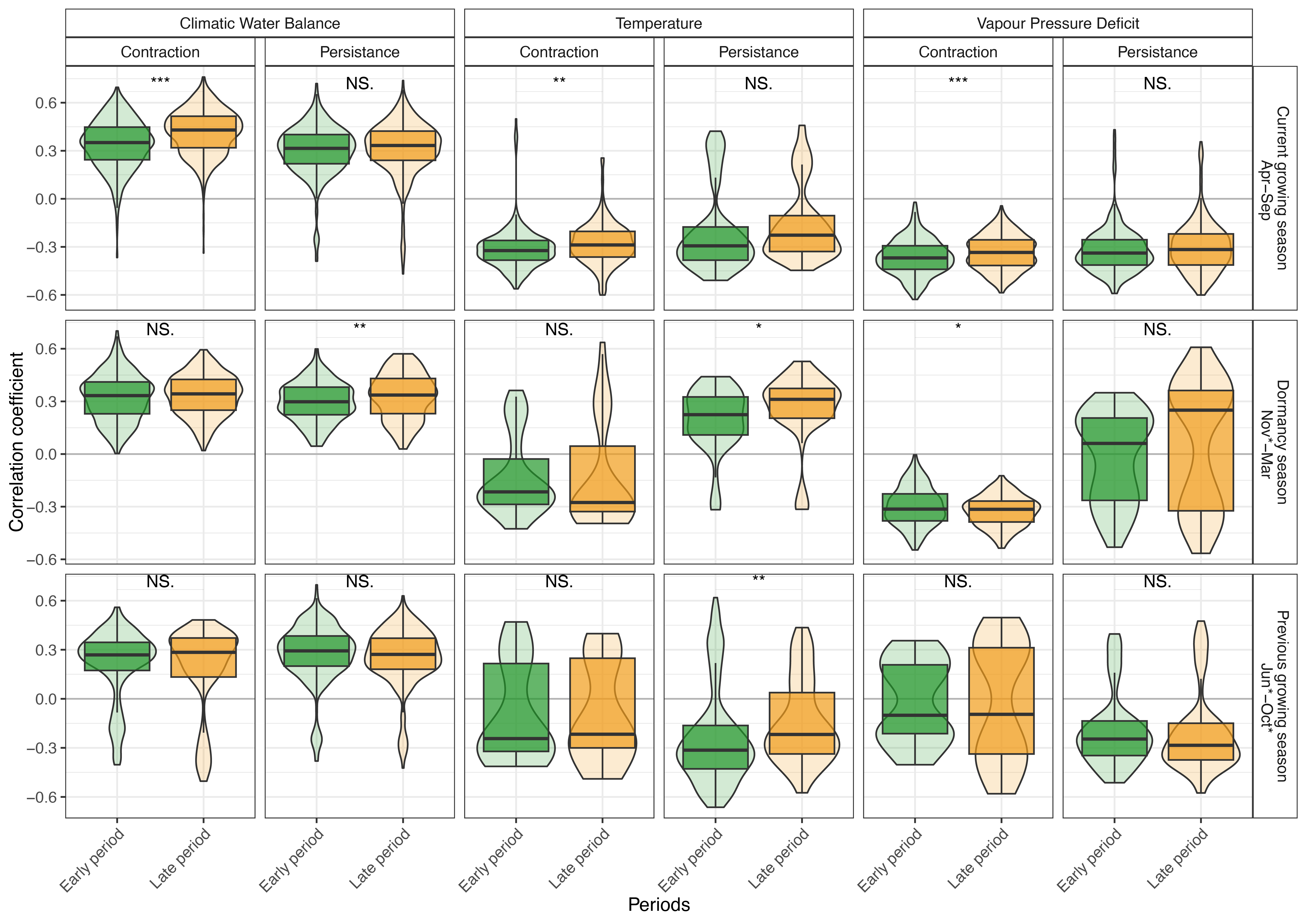


Figure S10 Correlation coefficients in early and late periods for intervals (months or cumulation of months) that recorded a significant correlation in the entire period; SDM projection related to the SSP370 scenario; annotation shows results from the Wilcoxon test, *** represents *p* < 0.001, ** represents *p* < 0.01, * represents *p* < 0.05, N.S. represents *p*  > 0.05.

Table S3 Summary of the optimized mixed-effects model for the sensitivity of oak based on correlations in the early / late periods with the climate factor as a fixed effect

| *Fixed effects* | $\beta$ | *CI* | *t* | *p* |  |
| --- | --- | --- | --- | --- | --- |
| (Intercept) | 0.296 | 0.197, 0.396 | 5.860 | **<0.001** |  |
| bio18 | -0.007 | -0.011, -0.003 | -3.611 | **<0.001** |  |
| bio9 | 0.000 | -0.004, 0.004 | 0.040 | 0.968 |  |
| bio10 | -0.243 | -0.250, -0.237 | -77.468 | **<0.001** |  |
| bio11 | -0.301 | -0.307, -0.295 | -95.772 | **<0.001** |  |
| Climatic parameter [tmed] | 0.029 | 0.024, 0.034 | 11.415 | **<0.001** |  |
| Climatic parameter [vpd] | 0.000 | -0.000, 0.000 | 0.831 | 0.406 |  |
| SDM projection [persistence] | 0.098 | 0.090, 0.107 | 22.744 | **<0.001** |  |
| period [late] | 0.114 | 0.106, 0.123 | 26.412 | **<0.001** |  |
| age | -0.004 | -0.011, 0.003 | -1.073 | 0.283 |  |
| Climatic parameter [tmed] × SDM projection [persistence] | -0.007 | -0.011, -0.003 | -3.611 | **<0.001** |  |
| Climatic parameter [vpd] × SDM projection [persistence] | 0.000 | -0.004, 0.004 | 0.040 | 0.968 |  |
| period [late] × SDM projection [persistence] | -0.243 | -0.250, -0.237 | -77.468 | **<0.001** |  |
| *Random Effects* | | | | | |
| σ^2^ | 0.035 | | | |  |
| τ_00_ _cod_ | 0.001 | | | |  |
| τ_00_ _seasons_ | 0.001 | | | |  |
| ICC | 0.047 | | | |  |
| No. corr. / No. plots / No. seasons | 45414/ 150 / 3 | | | |  |
| R^2^m / R^2^c | 0.241 / 0.277 | | | |  |

The response variables are correlation coefficients between RWI and climatic factors. The fixed part of the model included latitude (LAT), longitude (LON), BIO18 (mean monthly precipitation amount of the warmest quarter), BIO9 (mean daily mean air temperatures of the driest quarter), BIO10 (mean daily mean air temperatures of the warmest quarter), BIO11 (mean daily mean air temperatures of the coldest quarter), mean age of the stands (age), periods (early, late) and SDM projection (contraction, persistence) as categorical variables, and interaction between periods and SDM projections. The random part of the model included the site and the month nested in the season. Values represent the estimates of regression coefficients (β), 95% confidence intervals (CI), the *t* statistic, and the associated p-value of significance (bold values stand for significant fixed effects, *p* < 0.05). σ^2^ represents the variance of residuals, τ variance caused by random effects, ICC intra-class correlation coefficient R^2^_m_ is the marginal R^2^, and R^2^_c_ is the conditional R^2^.


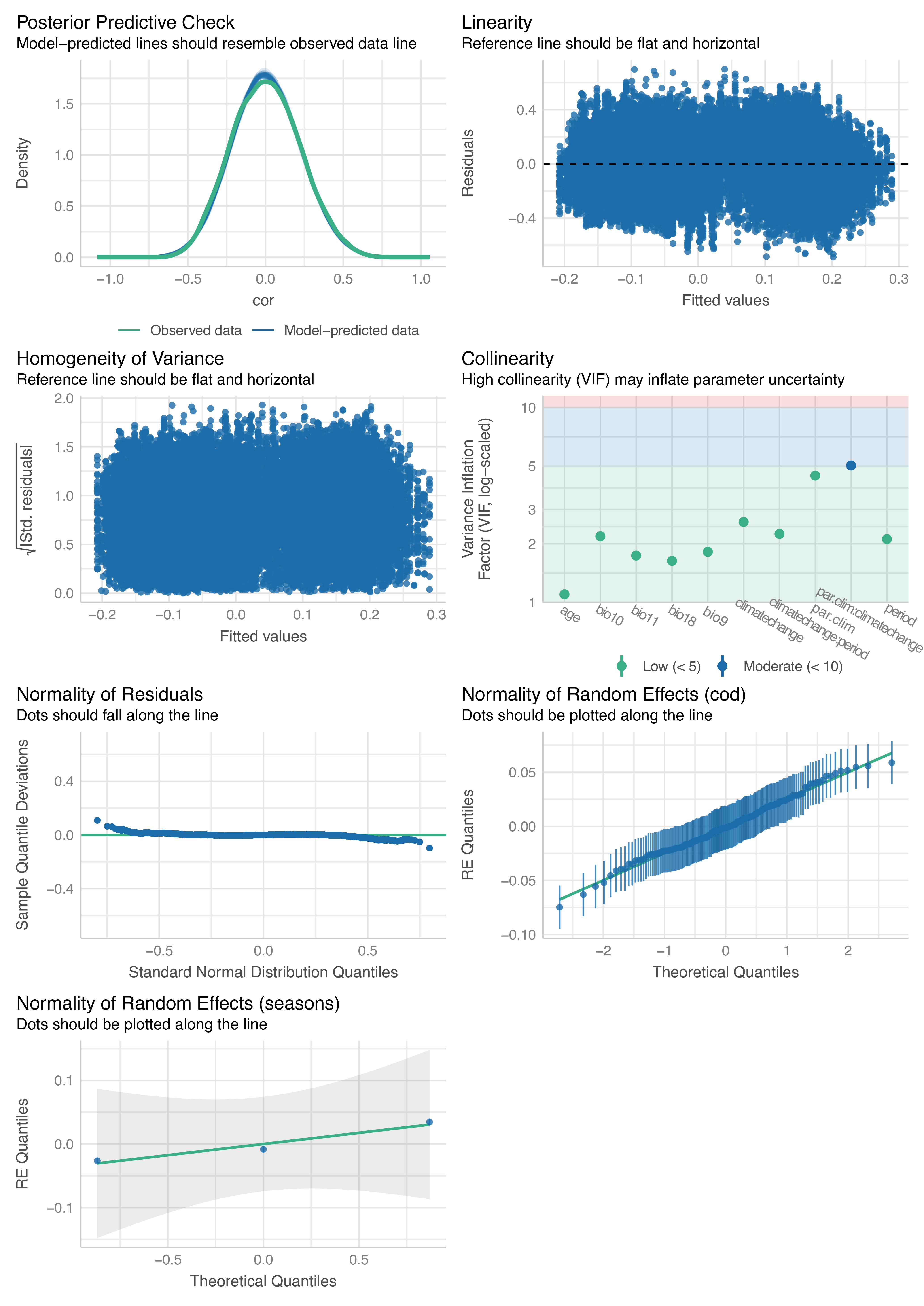


Figure S11 Diagnostic plots for the model presented in Table S3. Figure was generated using the *check_model()* function from ‘performance’ R package (Lüdecke et al., 2021)


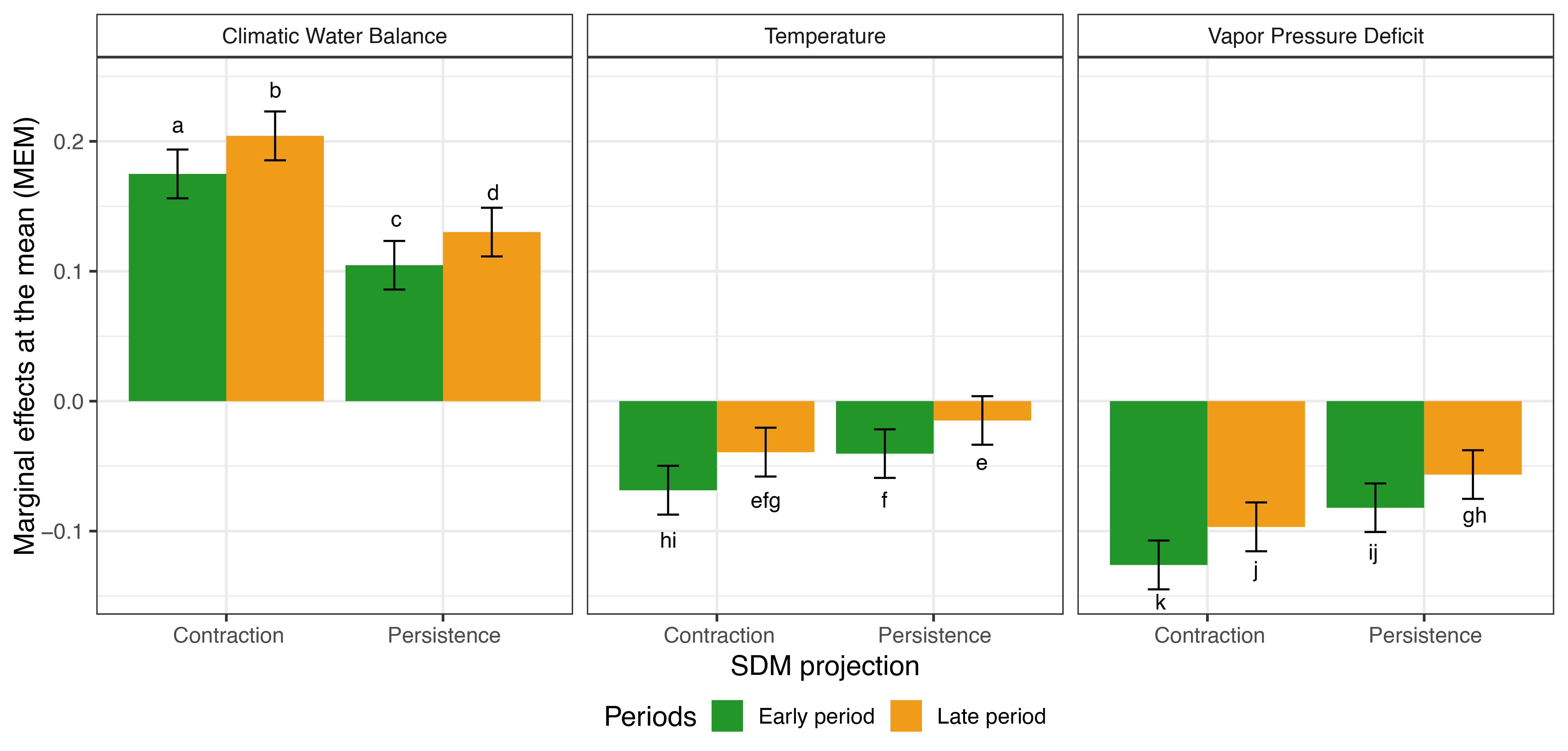


Figure S12 Marginal effects at the mean of study periods (i.e., early and late periods) on oak sensitivity to climate (Climatic Water Balance; Temperature; Vapor Pressure Deficit) in relation to SDM projection (potential range contraction or persistence); see Supplementary Table 3; error bars show ± one standard deviation; the letters show the significant differences between the categorial predictors according to Tukey test
